# Directional RNA-seq reveals highly complex condition-dependent transcriptomes in *E. coli* K12 through accurate full-length transcripts assembling

**DOI:** 10.1186/1471-2164-14-520

**Published:** 2013-07-30

**Authors:** Shan Li, Xia Dong, Zhengchang Su

**Affiliations:** 1 Department of Bioinformatics and Genomics, College of Computing and Informatics, The University of North Carolina at Charlotte, 9201 University City Blvd, Charlotte, NC 28223, USA; 2 Eastern Bee Research Institute, College of Food Science, Yunnan Agricultural University, Kunming, Yunnan, P. R. China

**Keywords:** RNA-seq, Prokaryote, *E. coli*, Transcriptome, Assembly, Transcription start site, Alternative operon, Antisense RNA, Non-coding RNA

## Abstract

**Background:**

Although prokaryotic gene transcription has been studied over decades, many aspects of the process remain poorly understood. Particularly, recent studies have revealed that transcriptomes in many prokaryotes are far more complex than previously thought. Genes in an operon are often alternatively and dynamically transcribed under different conditions, and a large portion of genes and intergenic regions have antisense RNA (asRNA) and non-coding RNA (ncRNA) transcripts, respectively. Ironically, similar studies have not been conducted in the model bacterium *E coli* K12, thus it is unknown whether or not the bacterium possesses similar complex transcriptomes. Furthermore, although RNA-seq becomes the major method for analyzing the complexity of prokaryotic transcriptome, it is still a challenging task to accurately assemble full length transcripts using short RNA-seq reads.

**Results:**

To fill these gaps, we have profiled the transcriptomes of *E. coli* K12 under different culture conditions and growth phases using a highly specific directional RNA-seq technique that can capture various types of transcripts in the bacterial cells, combined with a highly accurate and robust algorithm and tool TruHMM (http://bioinfolab.uncc.edu/TruHmm_package/) for assembling full length transcripts. We found that 46.9 ~ 63.4% of expressed operons were utilized in their putative alternative forms, 72.23 ~ 89.54% genes had putative asRNA transcripts and 51.37 ~ 72.74% intergenic regions had putative ncRNA transcripts under different culture conditions and growth phases.

**Conclusions:**

As has been demonstrated in many other prokaryotes, *E. coli* K12 also has a highly complex and dynamic transcriptomes under different culture conditions and growth phases. Such complex and dynamic transcriptomes might play important roles in the physiology of the bacterium. TruHMM is a highly accurate and robust algorithm for assembling full-length transcripts in prokaryotes using directional RNA-seq short reads.

## Background

In prokaryotes, several adjacent genes on the same strand of DNA are often co-transcribed as a polycistronic mRNA, forming a multi-gene transcription unit called an operon. Furthermore, in addition to protein- and RNA-coding genes, some parts of a non-coding sequence and the opposite strand of a coding sequence can also be transcribed under certain conditions, generating non-coding RNAs (ncRNAs) [1,2] and anti-sense RNAs (asRNAs) [3,4], respectively. Accumulating body of evidence suggest that ncRNAs [1,2] and asRNAs [3,4] may play important roles in the physiology of prokaryotes. Therefore, a full understanding of the transcriptomes of prokaryotic cells is necessary to annotate the functional elements in their genomes and to reconstruct the gene transcriptional networks in their cells. However, experimental determination of operon structures, ncRNAs and asRNAs by traditional molecular biology methods is time-consuming and labour-intensive. As a result, no single prokaryote has so far had all of its operon structures, ncRNA and asRNAs characterized using such methods. For instance, even for the most well-studied model bacteria *E. coli* K12 and *B. subtilis*, only 3,409 [5] and 736 [6] operons have been determined in their genomes using these methods, respectively, after decades of research while not each of their genes has been assigned to an operon. On the other hand, although a great progress has been made in computational prediction of operons [7-14] and small RNA genes [15-18], the accuracy of these predictors is still low [13,19], and they can only predict the static longest possible operons without considering possible alternative operons [7-14].

In the past few years, increasing applications in prokaryotes of whole genome directional (strand-specific) tiling array and directional RNA-seq techniques have completely changed our way to study and our view of the architecture and complexity of prokaryotic transcriptomes (for a thorough review, see [20-22]). For example, using a combination of whole genome directional tiling array and RNA-seq techniques, Guell *et al.*[23] found that operon utilizations in the reduced parasitic *M. pneumoniae* genome were highly variable and dynamic, almost half of the 139 identified multi-gene operons showed varying levels of (dynamic) expression in a staircase-like manner. Under different conditions, large operons could be transcribed as smaller sub-operons, resulting in many alternative transcripts, suggesting that the operon structures in *M. Pneumonia* were highly complex and dynamic, a phenomenon that was comparable to the alternative splicing in eukaryotes [23]. They also identified a large number of ncRNAs and asRNAs expressed under various culture conditions, hence a much larger portion of the genome was transcribed than originally anticipated [23]. Similar results were observed in many other taxonomically distinct species, such as *epsilon proteobacteria H. pylori*[24]; *firmicutes B. sutiblis*[25] and *B. anthracis*[26]; *cyanobacteria Synechocystis sp.* PCC6803 [27]; *euryarchaeota Halobacterium salinarum* NRC-1 [28]; and *bacteroidia Porphyromonas gingivalis*[29], to only name a few. However, not all these surprising observations were noted in some other studies. For instance, prevalent alternative operon utilizations were not reported in many studies in a variety of prokaryotes, such as *B. subtilis*[30], *Salmonella entericaserovar* Typhi [31], *Burkholderia cenocepacia*[32], *Caulobacter crescentus*[33], *Staphylococcus aureus*[34], *Vibrio cholera*[35], *Chlamydia trachomatis*[36], *Chlamydia pneumonia*[37], *Clostridium beijerinckii* NCIMB 8052 [38], *Listeria monocytogenes*[39], *Anabaena sp.* strain PCC 7120 [40], *Synechococcuselongatus* PCC 7942 [41], and *Sulfolobus solfataricus* P2 [42]. Contradictory results have also been reported. For instance, although Rasmussen *et al.*[30] did not note alternative operon utilizations in *B. subtilis*, more recently, Nicolas *et al.*[25] observed highly prevalent condition-dependent operon utilizations using a similar tiling array technique. Moreover, although most of these studies found extensive asRNA and ncRNA transcriptions, the levels of their prevalence could vary quite differently from different studies even in the same strains. For instance, although Selinger *et al.*[43] reported that up to 4,000 *E. coli* K12 genes had asRNA transcriptions using directional tilling arrays, Dornenburg *et al.*[44] only identified about 1,000 asRNAs in the same strain under similar growth conditions using directional RNA-seq. These discrepancies can be due to different experimental conditions and methods used in these studies. Nevertheless, they inevitably raise the question: are the prevalent alternative operon utilizations, asRNA and ncRNA transcriptions ubiquitous phenomena in all prokaryotes or only prevalent in some specific species?

*E. coli* K12 is probably the best known free living model organism [45,46], where novel biological hypotheses and computational algorithms can be tested. Indeed, it is mainly through the studies in *E. coli* K12 that we have understood many fundamental biological processes, including the mechanisms of gene transcriptional regulation [47-49]. As a result, the *E. coli* K12 genome is in fact the best understood among all the model organisms in almost all aspects [50,51]. Since the finishing of its genome sequence in 1997 [52], almost all newly developed high throughput technologies have been applied to this bacterium. As a result, 4,501 genes have been experimentally or computationally identified in the MS1655 strain, and 3,384 (75%) of them have been assigned a biochemical function [51]. Of these 3,384 genes with an assigned function, 2,941 (87%) had their functions characterized experimentally (66% of the total encoded genes) [46,51]. The products of the 918 genes with experimentally characterized function catalyze 1,008 metabolic reactions, which constitute the best understood metabolic network [51]. As for its transcriptomes and transcriptional regulatory networks, RegulonDB database [53] that is dedicated to compiling all experimentally verified relevant information in *E. coli* K12 has documented 3,409 operons (including singleton genes), 1,878 promoters, 1,940 transcription factor binding sites of 175 transcription factors (TF) in the regulatory region of 703 operons, and 2,697 TF-target gene regulations [53]. Furthermore, more than a hundred of ncRNAs and asRNAs have been experimentally identified in the *E. coli*[54-56]. More recently, Cho *et al.*[57] applied a combination of tiling array, 5’-end RNA deep sequencing, RNAP ChIP-chip and proteomics analyses to reveal the transcription unit architecture in the *E. coli* K12 genome. They identified 4,661 transcription units, many alternative Transcription Start Sites (TSSs), alternative operons and ncRNAs under a few cultural conditions. In another study, Mendoza-Vargas *et al.*[58] identified ~1,500 new TSSs using a modified 5’-RACE method and a 5’-end RNA sequencing method in the genome. Consequently, after more than 40 years intensive molecular genetics research in this bacterium, including the recent high throughput studies [43,44,57,58], our experimentally validated knowledge of the transcriptome and gene regulatory systems in *E. coli* K12 is the most complete currently available for any organism [46,51]. However, ironically, our understanding about the complexity of the transcriptomes in this model bacterium is rather limited compared to its counterpart model Gram-positive bacterium *B. subtilis*[25]. In particular, large scale dynamic and alternative operon utilizations under various conditions have not been reported in *E. coli* K12, so do they exist in this bacterium? Furthermore, how many asRNAs and ncRNAs are transcribed in *E. coli* K12 given the aforementioned inconsistent results [43,44]?

Technically, compared to directional tiling array techniques, directional RNA-seq methods are more suitable and powerful tools for understanding the complexity of the prokaryotic transcriptomes due to their single-nucleotide resolution, higher dynamic range, and lower noise levels, thus they have gained increasing popularity [59]. One important step in RNA-seq data analysis is to accurately assemble all meaningful transcripts in their full-length, so that correct conclusions can be drawn from tens of thousands of RNA-seq short reads generated by next generation sequencing (NGS) technologies. However, it has been recently released [23,24,28,29,60,61] and we will indicate later in this paper, that the coverage of reads generated by the current RNA-seq techniques on transcribed regions is highly non-uniform. More seriously, there are even numerous uncovered parts in transcribed regions, leading to gaps in otherwise a continuous mapping in the region [62-67]. These highly non-uniform coverage and uncovered gaps make the transcripts assembly and quantitative analyses highly challenging tasks [23,60,68-71]. Several technical problems in the current RNA-seq library construction protocols and sequencing technologies have been identified responsible for the non-uniform coverage and gaps. First, the chemical RNA fragmentation methods used in many protocols may have a bias to break or degrade some sequences [72]. Second, the random primer based reverse transcription may preferentially transcribe some sequences [66,73]. Third, ligases may preferentially link the adaptors to some sequences [74-76]. Fourth, PCR amplification is well-known for introducing GC content-dependent bias in libraries [77-80]. Fifth, it was recently found that sequencing errors could be biased to some specific sequences, making such sequences missing from the reads [81]. Moreover, prokaryotic RNAs are more labile than their counterparts in eukaryotes, thus segments of some RNAs can be more easily lost during the library preparation. Although some of these problems can be avoided by new technical development, such as using FRET-seq for amplification-free sequencing to avoid GC content-dependent PCR bias [82], or using single RNA molecular sequencing for longer reads to ease the assembly problem [83,84], no effective routine technique has yet been developed to avoid all these problems.

On the other hand, although several transcriptome assemblers using RNA-seq short reads have been developed in the past few years, they are mainly for reconstructing alternative isoforms in eukaryotes [70]. These assemblers can be classified into two basic categories [70]: the reference-based assemblers when a reference genome sequence is used, and the *de novo* assemblers when a reference genome is not used. The reference-based assemblers usually involve two steps: RNA-seq reads are first mapped to the reference genome using an aligner, such as BLAT [85], TopHat [86] or Bowtie [87], and then a graph representing all possible isoforms from overlapping reads is constructed, and the isoforms are resolved by traversing the graph. Examples of this strategy include Cufflinks [71] and Scripture [88]. The *de novo* assemblers such as Trinity [89], Oases [90], TransAByss [91], Rnnotator [92], and Multiple-k [93], generally assemble isoforms based on a De Bruijn graph constructed using overlapping reads. The advantage of *de novo* strategy is that it can assemble transcripts when a reference genome is not available and can recover transcripts that are missing in the genome assembly. However, *de novo* transcriptome assembly is very sensitive to sequencing errors, in particular, missing and chimerical reads in the dataset, thus their accuracy is generally lower than the reference-based approaches [70].

*De novo* transcriptome assembly in prokaryotes can also be more challenging in prokaryotes owing to the prevalence of uncovered gaps caused by the aforementioned technical reasons and the unique prosperities of their RNAs. Fortunately, with thousands of sequenced prokaryotic genomes available now, transcriptome assembly in prokaryotes can often be done using the reference-based approaches. However, the only reference-based transcriptome assembler for prokaryotes that we are aware of is a Hidden Markov Model (HMM)-based method for reconstructing operons in *Bacillus anthracis*[94], yet no tool was delivered from this research. Furthermore, there are at least two limitations in this method. First, the prevalently uncovered gaps were not explicitly treated in this method [94], thus the interrupted partial transcripts could not be effectively bridged. Second, although this method attempted to model transcripts of different transcription levels using different expression states, it did not allow transitions among the states [94]. Thus, without an effective method to correct the high non-uniformity of the read coverage along a transcript [65,72,73,75,81], this method can break a transcript into smaller fragments. Because of the lack of a good prokaryotic assembler, currently prokaryotic transcripts were assembled by either simply stitching the two covered segments if the gap between them is shorter than a cutoff [26], or determining 5’ and 3’ ends of transcripts via a probability-based approach [41], or relying on an additional source of information for the assembly, such as tiling array data that tend to have a more even and consecutive coverage along transcribed regions albeit at lower resolution [23,25]. As RNA-seq becomes a routine technique for probing transcriptomes in prokaryotes, an efficient and accurate full-length transcripts assembly algorithm and tool tailored to prokaryotes are urgently needed in the research community.

To gain a better understanding of the complexity of the transcriptomes in *E. coli* K12, we have profiled the transcriptomes of the bacterium under different culture conditions and growth phases using a highly specific directional RNA-seq technique that can capture various types of transcripts in the cells, including mRNAs, asRNAs, and ncRNAs. To assemble all types of full length transcripts using the directional RNA-seq short reads, we have developed a new Hidden Markov Model based algorithm, TruHMM (TRancription Unit assembly by a Hidden Markov Model), attempting to addresses the highly non-uniform read coverage and uncovered gap problems of current RNA-seq techniques. TruHMM differs from the earlier HMM-based algorithm [94] in several ways (for details see Methods and Discussion). In particular, TruHMM overcomes the aforementioned limitations of the earlier method by allowing a transcript to have highly non-uniform coverage at different positions, and explicitly addressing the uncovered gap problem using a sliding window-based centroid read counting strategy in a pre-processing step. Furthermore, TruHmm can also predict alternative operons and TSSs of the assembled transcripts. When evaluated on sets of known operons, asRNAs and ncRNAs in *E. coli* K12, TruHMM was able to assemble various types of transcripts with rather high accuracy. The parameters trained in *E. coli* K12 can be applied to an earlier directional RNA-seq dataset in *H. pylori*[24] with similarly high accuracy, and vice versa, thus TruHMM is also very robust. Based on the transcripts assembled in TruHMM, we found that 46.9 ~ 63.4% of expressed operons were utilized in their putative alternative forms, 72.23 ~ 89.54% open reading frames had putative asRNA transcriptions and 51.37 ~ 72.74% intergenic regions had putative ncRNA transcriptions under different culture conditions and growth phases. Thus, it seems that there are more prevalent alternative operon utilizations as well as asRNA and ncRNA transcriptions in *E. coli* K12 than originally anticipated, and they may play important roles in the physiology of the bacterium.

## Results

### Our directional RNA-seq libraries are highly strand-specific and can capture various types of RNAs

**Figure 1:**
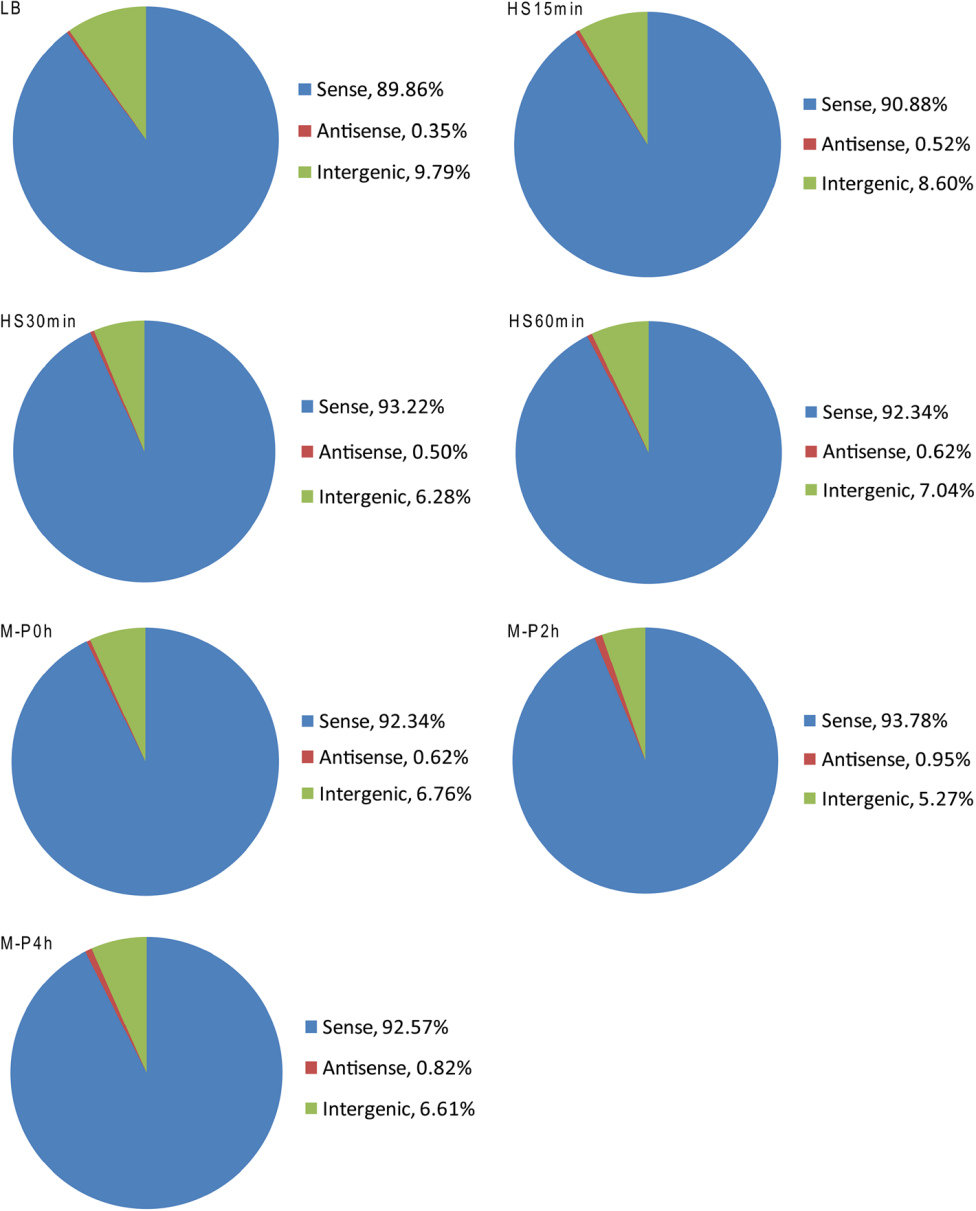
**Strand specificity of the directional RNA-seq libraries.** The percentage of total nucleotides mapped to sense strand, antisense strand and intergenic regions is shown for the seven samples.

We prepared the directional RNA-seq libraries from seven *E. coli* K12 samples collected at the log phase growth in LB, and different time points under heat shock (HS) or phosphorus starvation (M-P) treatments, denoted as LB, HS15 min, HS30 min, HS60 min, M-P0 h, M-P2 h, and M-P4 h to reflect the treatment and sampling time point. The experimental procedure of our work is listed in Additional file 1: Figure S1. The libraries were sequenced on either the Illumina GAII or the HiSeq 2000 platform. Specifically, the sample LB was sequenced using the GAII platform, samples HS30 min, HS60 min, M-P0 h, and M-P2 h were sequenced using the HiSeq 2000 platform, whereas samples HS15 min and M-P4 h were sequenced using both the platforms. Each sample sequenced using the HiSeq 2000 platform was repeated twice (technical replicates). The reads obtained from different platforms for the same sample are highly correlated (Additional file 1: Figure S2), thus the data for the same sample were combined for the analysis. A total of 330,611,663 reads were generated from the seven samples. The mapping statistics of the samples are summarized in Additional file 1: Table S1 showing that 23.07 ~ 44.18% of reads could be uniquely mapped to the genome, resulting in 7,735,369 ~ 29,581,761 uniquely mapped reads in each sample, corresponding to a sequencing depth of 93 ~ 355 times of the genome. Of the 47.08 ~ 63.04% multiple mapped reads in each sample, over 99.6% were from duplicated tRNA/rRNA genes (data not shown). Thus discarding these multiple mapped reads does not affect our analysis of mRNA, asRNA and ncRNA transcriptions. Furthermore, as shown in Figure 1, in all the samples over 90% and less than 10% of the total mapped nucleotides were mapped to the sense strand and intergenic regions, respectively, with only 0.35 ~ 0.95% of the total mapped nucleotides mapped to the antisense strand. Moreover, as shown in Additional file 1: Figure S3, our uniquely mapped reads consisted of well-balanced different sizes of RNA insertions, indicating that, in additional to mRNA, our library preparation protocol could potentially capture small RNA species such as asRNAs and ncRNAs, which were otherwise left out by a typical size selection step in other library preparation protocols. All these results indicate that our sequence reads are highly strand-specific and of high quality, which is consistent with an earlier result using a similar library construction protocol [61]. The seven sequence datasets have been submitted to the Gene Expression Omnibus (GEO) database with accession number GSE48151.

### Uncovered-gaps in transcribed regions are prevalent and read coverage is highly non-uniform

**Figure 2:**
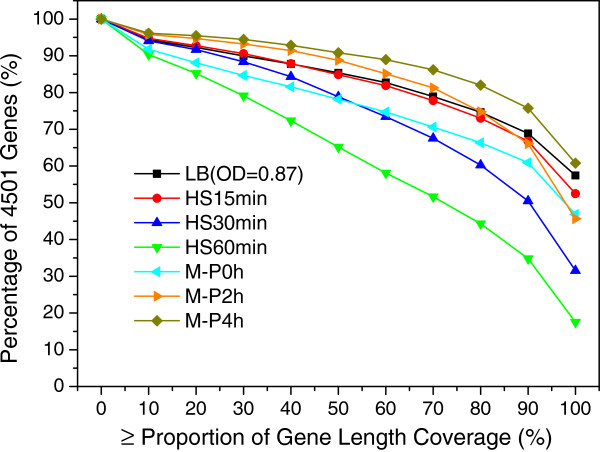
**Distribution of the genes with more than the indicated percentage of their length covered by at least one read in the samples.** Less than 60% of genes have their length completely covered by at least one read. Over 80% genes have over 50% of their length covered by at least one read except for sample HS60 min.

**Figure 3:**
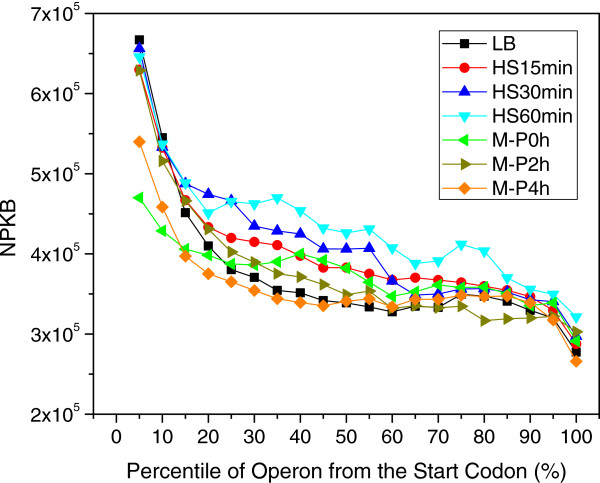
**Reads are biased to the 5’-end of operons.** The sufficiently expressed known multiple-gene operons (Additional file 2) and singleton operons are equally divided into 20 bins, and the average expression values in each bin of all operons in each sample were displayed. The top 10% most highly expressed genes were excluded from the calculation.

However, as shown in Figure 2, even with such deeply sequencing coverage, less than 60% genes in the genome had their length completely covered by at least one read, while only less than 90% genes in the genome had at least 10% of their length covered by at least one read, suggesting that some transcribed regions were not covered by the reads, leaving uncovered gaps in transcribed regions. The same problem has been widely noted in both eukaryotes [61-63,66,67,95] and prokaryotes [24,60] due to the aforementioned technical artefacts of the current RNA-seq techniques [65,72,73,75,81]. In fact, we found that this uncovered gap problem was even more serious in many published prokaryotic datasets we have reanalyzed, a typical example from [60] is shown in Additional file 1: Figure S4. These prevalent uncovered gaps may be also partially caused by the loss of some RNA fragments during the library preparation due to the highly labile nature of prokaryotic RNAs as mentioned earlier. Our data seems to support this hypothesis, as the percentage of gene body coverage in our samples collected under heat shock treatment were generally lower than that in other treatments, in particular, after 30 and 60 min heat shock (Figure 2). It is well known that RNAs have a shorter living time at a higher temperature. It is because of this uncovered gap problem that we define a gene with ≥50% of the length covered by at least one read to be sufficiently expressed. Also, this 50% cutoff was chosen, as all the samples except HS60min had over 80% of genes with at least 50% length being covered (Figure 2). Additionally, as shown in Figure 3, our libraries were also biased to the 5’-end of transcription units, which is consistent with the earlier results [24,57,58].

Furthermore, we also found that the read coverage along genes were highly non-uniform (an example is shown in Additional file 1: Figure S5). Interestingly, the pattern of non-uniform coverage did not depend on the culture conditions and growth phase; rather, it strongly depended on the positions in the transcribed region (Additional file 1: Figure S5). Such highly non-uniform read coverage along a transcribed region has been widely noted in recent studies [23,24,28,29,60,61], and were shown to be caused by several technical artifices in current RNA-seq techniques [66,72-81]. Clearly, both the uncovered gaps and highly non-uniform read coverage along transcribed regions make the full-length transcript assembling and alternative operon identification challenging tasks.

### TruHMM assembles operons with high accuracy

**Figure 4:**
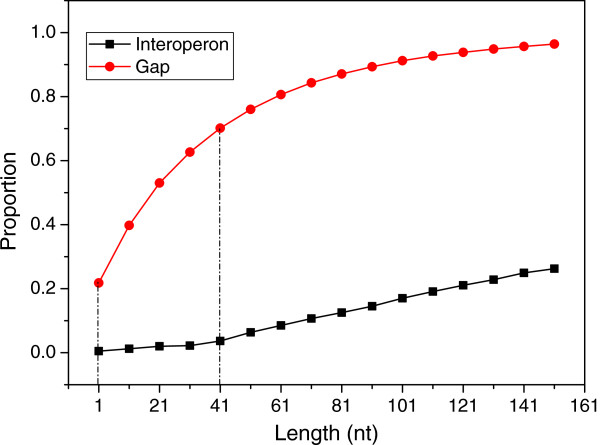
Cumulative distributions of the length of interoperonic regions and the length of gaps in sufficiently expressed regions.

**Figure 5:**
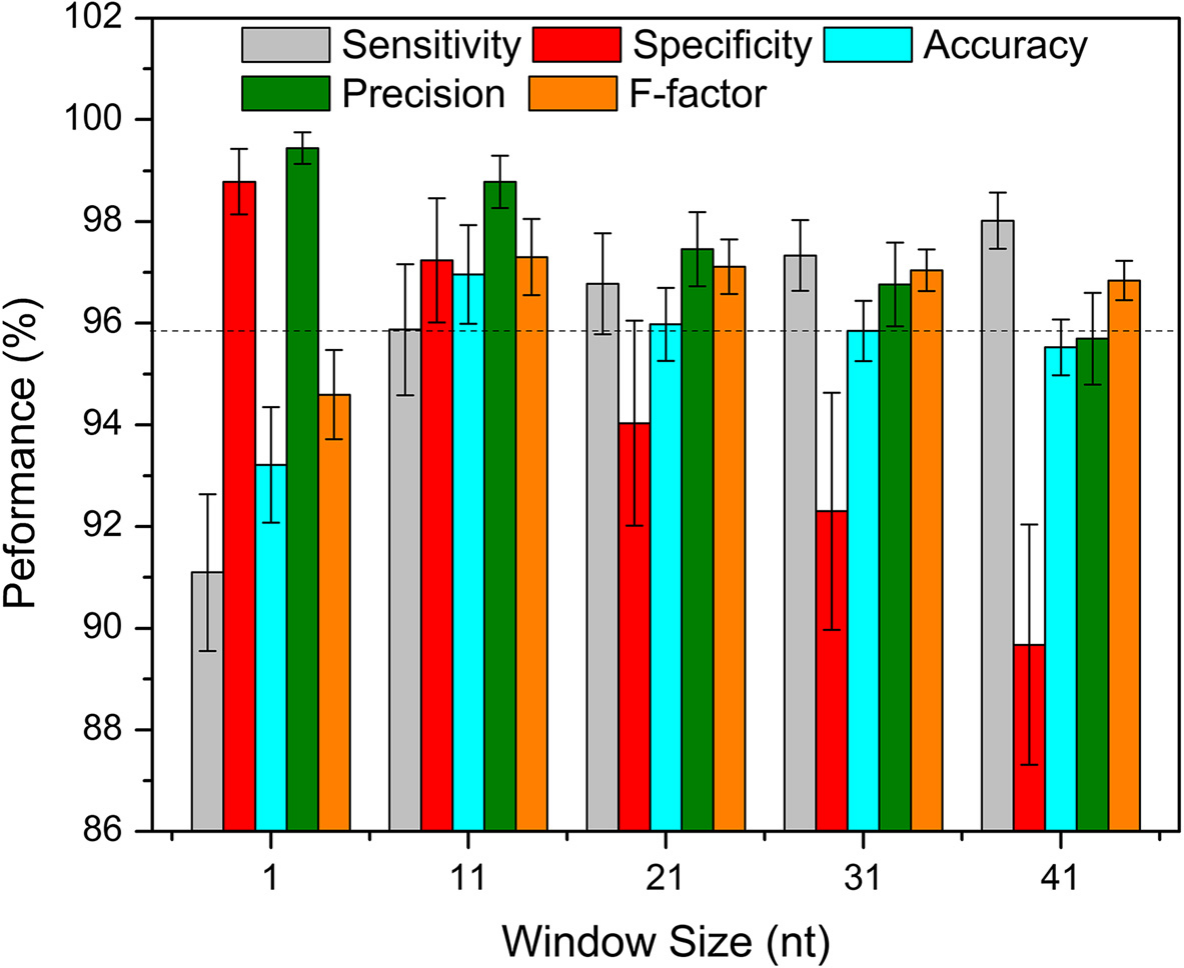
**Evaluation of the algorithm based on operon pairs in the seven samples.**The dashed horizontal line is at the 95.87% level, and the vertical bars indicate standard errors.

**Figure 6:**
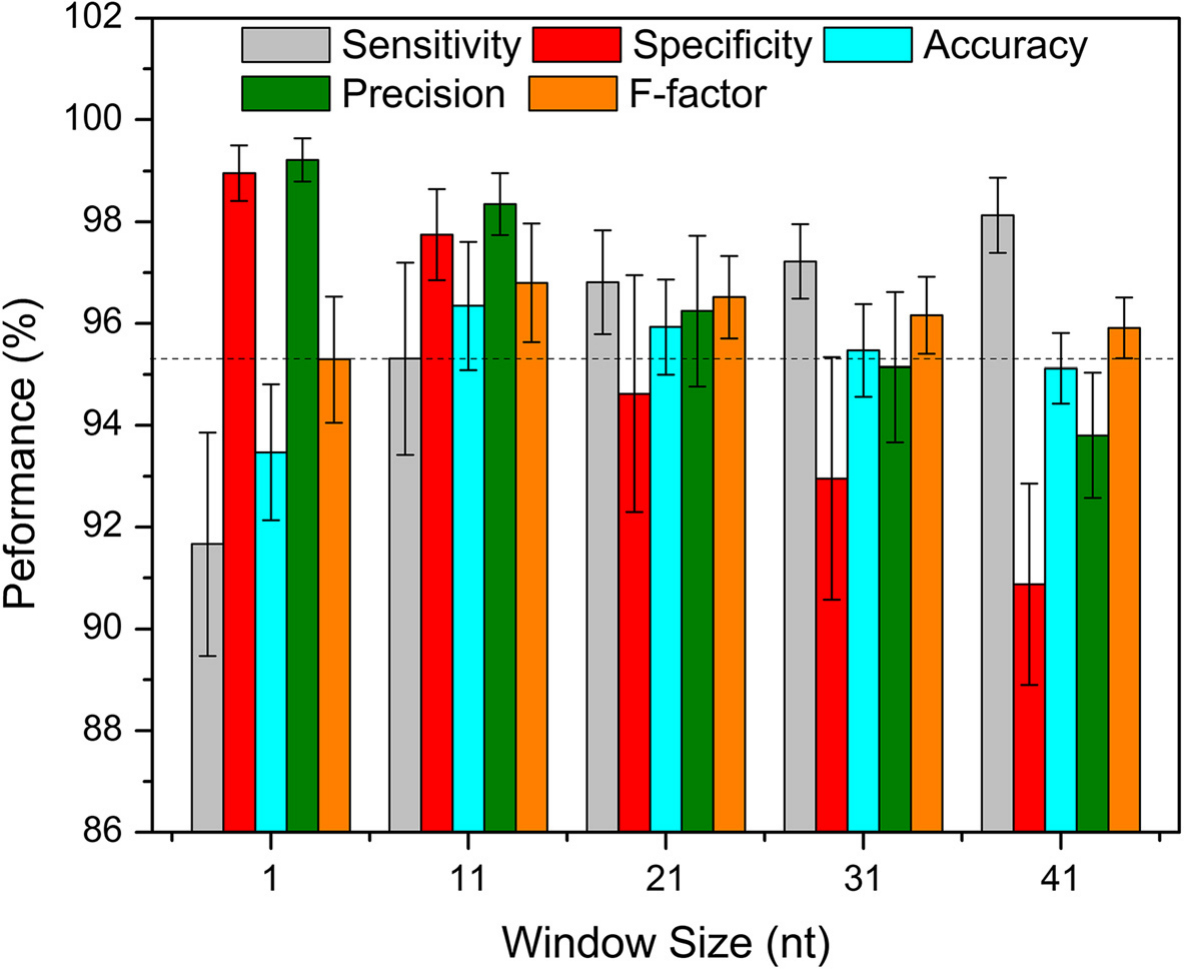
**Evaluation of the algorithm based on entire operon structures in the seven samples.** The dashed horizontal line is at the 95.3% level, and the vertical bars indicate standard errors.

We used the 476 experimentally verified operons in RegulonDB (Additional file 2) to train the parameters of the HMM and applied the leave-one-out strategy to test our TruHMM algorithm. To compensate for the negative effect of uncovered gaps in the expressed regions on assembling, we used a centroid coverage value in a sliding window to represent the reads coverage for each nucleotide of DNA (see Methods). Meanwhile, we do not want to increase false positives by mistakenly bridging irrelevant reads using such a strategy. To find an appropriate widow size for this purpose, we plotted the distributions of interoperonic and gap lengths shown in Figure 4, which suggest that the optimal window size might be shorter than 41 nt. Therefore, we evaluated the performance of our algorithm when the window size varied from 1 to 41 nt with an increment of 10 nt on all the seven samples using the leave-one-out validation strategy (Methods). As shown in Figure 5, when evaluated using the adjacent operon pairs (neighbouring gene pairs within an operon, for details see Methods), our algorithm was very robust for the choice of the window size in the range of 11 ~ 21 nt (the mean values for each metric are ≥ 94%). Particularly, when the window size *L* = 11 nt, the algorithm achieved probably the best-balanced performance (the mean values for each metric are ≥ 95.87%), especially in terms of the three most important measures: sensitivity, specificity and accuracy. When evaluated using the entire operon structure, our algorithm still achieved very good performance with all the five metrics being over 94.6% for window size of 11 ~ 21 nt (Figure 6), and the best performance (the mean values for each metric are ≥ 95.3%) was also obtained when *L* = 11 nt. Therefore, we chose *L* = 11 nt for our further analysis. We also evaluated the effect of sequencing depth on the performance of our algorithm. As shown in Additional file 1: Table S2 using M-P4 h as an example, when the sequencing depth is over 153 times of genome size, our algorithm was very robust to the sequencing depth.

### The performance of TruHMM is robust

To evaluate the performance of TruHMM and the robustness of its parameters on different organisms and datasets, we first applied TruHMM with the parameters trained on the *E. coli* K12 dataset to the earlier directional RNA-seq datasets of *H. pylori* generated under five different culture conditions [24]. We then trained the algorithm using an *H. pylori* training set (Additional file 3, and see Methods) based on the results in [24], and applied the algorithm with the trained parameters to both the *H. pylori* and *E. coli* K12 RNA-seq datasets. Remarkably, the operons reconstructed in both *H. pylori* and *E. coli* K12 using the *E. coli-* or *H. pylori*-trained parameters are exactly the same (data not shown), and have high accuracy measured by all the five metrics (Figures 5 and 6, and Additional file 1: Table S3 and S4). This might be explained by the fact that the parameters of the algorithm trained on the *H. pylori* training sets and on the *E. coli*K12 training sets are almost the same (Additional file 1: Table S5), although our *E. coli* and the earlier *H. pylori* RNA-seq datasets were generated by quiet different methods. These results unambiguously demonstrate that the performance of our algorithm is highly robust, thus parameters trained in one organism can be well extended to other organisms, at least in our tested datasets. The assembled operons in *H. pylori* for each sample are listed in Additional file 4.

### The boundaries of operons can largely be captured by our libraries and assembled by TruHMM

We next evaluated the ability of TruHMM to define operon boundaries, i.e., the TSSs and transcription termination sites (TTSs) of assembled transcripts. However, an accurate evaluation of predicted operon boundaries is complicated by the recently discovered fact that alternative TSSs and TTSs are far more prevalent than previously thought [23-25,57,58] and the lack of a gold standard TSS and TTS datasets because although some different TSSs and TTSs are documented for some operons in RegulonDB, they were generally characterized in different studies under various conditions that are not necessarily the same as we used in this study. Thus, we evaluated our reconstructed TSSs by the following alternative ways. First, we wanted to know how many experimentally verified TSS in RegulonDB could be recovered by the boundaries of our assembled operons in any of the seven samples. If two known TSSs in RegulonDB are within 10nt from each other, we considered them as the same one in our evaluation. Thus, there are 1,742 known TSSs (Additional file 5) associated with the genes transcribed in at least one of our seven samples. We considered a known TSS was recovered by our predicted TSS if they were at most 50nt from each other. Using this criterion, 908 out of 1,742 (~52.1%) known TSS were recovered by a total of our 5,706 predicted TSSs (Additional file 5). Second, as for the remaining 4,798 predicted TSSs with no match to a known TSS, 2,830 of which appeared in at least two samples, thus they are likely to be novel true TSSs. For example, although genes *b2628-b2627* on the reverse strand is documented as an operon in RegulonDB, there is no TSS documented for gene *b2628*. We predicted a potential TSS in the upstream intergenic region of *b2628* (2,763,486) in five samples (Additional file 1: Figure S5). The remaining 1,968 predicted TSSs appeared only in one sample. The 4,798 predicted TSSs are listed in Additional file 6. The low coverage of known TSSs in RegulonDB does not necessarily indicate the inaccuracy of our prediction, considering the prevalence of alternative TSSs utilizations under different conditions and the fact that TSSs in RegulonDB were mostly characterized by different researchers, and under different conditions. Therefore, the limited number of TSSs in RegulonDB might be the major reason.

**Figure 7:**
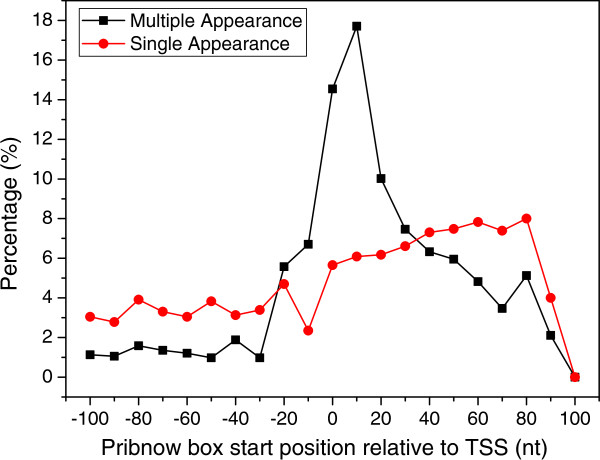
Distribution of the Pribnow box start position relative to predicted TSS appearing in multiple samples (black dots) or in a single sample (red dots).

Third, we checked whether there is a potential σ^70^ binding site (Pribnow box) near the predicted TSSs. To this end, we used the motif profile of the Pribnow boxes (Additional file 1: Figure S6A) found by MEME [96] in 539 (31%) out of 1742 upstream promoter sequences to scan for the potential Pribnow box in the [-100 nt, 100 nt] interval around the predicted TSSs. According to the distribution of the scanning scores in the random promoter sequences (see Methods), when a score is greater than 4.5487, the corresponding empirical p-value would be smaller than 0.05. In all, 1,327 (47%) out of the 2,830 predicted putative TSSs appearing in multiple samples harbour a putative σ^70^ binding site around predicted TSSs with a p-value ≤ 0.05 (Additional file 1: Figure S6B and Additional file 7), and 1,150 out of the 1,968 (58.4%) predicted putative TSSs appearing in only one sample bear a putative σ^70^ binding site with p-value ≤ 0.05 around the predicted TSSs (Additional file 1: Figure S6C and Additional file 7). However, the predicted TSSs appearing in multiple samples are more likely to be genuine ones since around 80% of which have a potential σ^70^ binding site located around the [-50 nt, 50 nt] interval of the predicted TSSs, compared to the rather evenly distributed Pribnow box positions of predicted TSSs appearing in a single sample (Figure 7).

Lastly, Sharma *et. al*[24] have determined 735 primary TSSs (defined as the most frequently used TSS by an annotated transcript, supplementary information of [24]) in *H. pylori*, using dRNA-seq technique that enriches the reads coverage on the 5’ end of a transcript. Therefore, the TSSs determined in this study could be a good dataset to test the accuracy of our algorithm. Specifically, we compared our predicted TSSs in *H. pylori* using their directional RNA-seq datasets with their TSSs determined by dRNA-seq. On average, 73.12% of our predicted TSSs in each sample are located within the [-50 nt, 50 nt] interval around a TSS determined by dRNA-seq (Additional file 1: Table S6). Thus our algorithm has achieved a rather high specificity. Our predicted TSSs in each of the five samples, located within the [-50 nt, 50 nt] interval around a verified TSS are listed in Additional file 4. Furthermore, we used the primary TSS to check the recall rate (sensitivity) of our program. Our program detected 558 (~76%) out of the 735 total primary TSSs. The majority of the verified TSSs recalled by our algorithm had a dominant coverage on the 5’ end of the transcript, one of such cases is shown in Additional file 1: Figure S7. By contrast, the majority of primary TSSs missed by our algorithm did not have a dominant read coverage on the 5’-end, two such cases are shown in Additional file 1: Figure S8. The primary TSSs both covered and missed by TruHMM are listed in Additional file 8. The much higher recovery rate of known TSSs by our algorithm in *H. pylori* than in *E. coli* K12 might be due to the fact that the gold standard dataset in *H. pylori* were derived from the same conditions as the RNA-seq datasets that we used for assembling the transcripts, while the datasets in RegulonDB were derived under various conditions.

As for the TTS predictions, our algorithm recovered 148 out of 221 (~67%) known TTSs associated with expressed genes in *E. coli* K12 (Additional file 5), which is higher than the recovery rate of known TSSs, even though the mapped reads are strongly biased to the 5’-ends (Figure 3). The lower recovery rates of known 5’ ends (TSS) compared to 3’ ends (TTS) might indicate that operons utilize more alternative TSSs than TTSs under different conditions. In other words, the predicted TSSs without a match with a known TSS in RegulonDB are likely to be novel alternative TSSs used in different conditions. Taken together, all these results strongly suggest that most of the predicted TSSs and TTSs are likely to be true transcription boundaries. The assembled operons and their alternative TSSs in each sample are listed in Additional file 9. However, as also demonstrated in earlier studies [24,57,58], to more accurately detect TSSs and TTSs of transcripts/operons, in particular TSSs, in addition to directional RNA-seq datasets, special datasets targeted to the 5’-endof transcripts are clearly needed, such as dRNA-seq data [24] and datasets for the more recently discovered transcription start site RNAs (tssRNAs) [97].

### Condition-dependent alternative operon utilizations appear to be prevalent in *E. coli* K12

**Figure 8:**
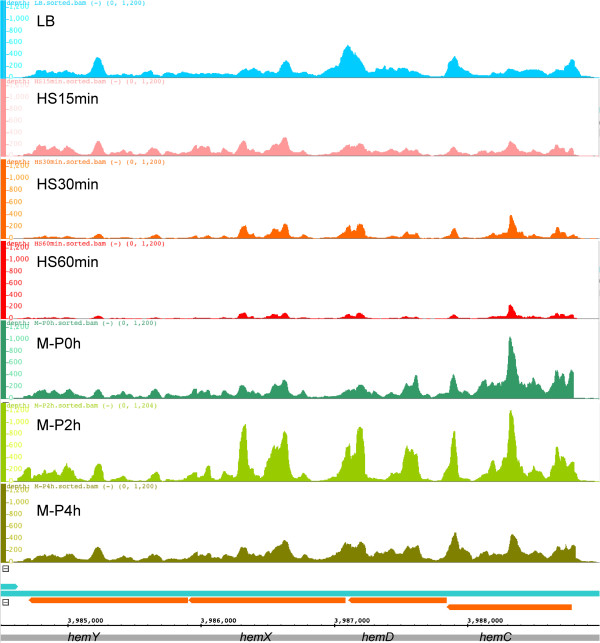
**Position-dependent non-uniform coverage of the reads along the *hem *operon *hemCDXY.*** The vertical axis is the number of reads covered at the positions. The orange and dark green bars at the button of the graph represent the reverse and forward strands, respectively. Segments with arrows represent genes. The graphs were generated using IGB. To make the expression levels for the four genes comparable in different samples, the same scale (1,200) of the vertical axis is used for all the samples. Although this four-gene operon was consecutively covered by the reads under different cultures and growth phases, there are highly similar patterns of position-dependent non-uniform coverage of the reads along the operon in the samples.

As summarized in Additional file 1: Table S7, our algorithm detected more than 2,000 operons involving more than 4,200 genes in each sample. There were 1,121 consistent operons that were transcribed in at least two of the seven samples, and 207 of which were multiple-gene operons (Additional file 10). Of these 207 consistent multiple-gene operons, 206 were expressed in all the seven samples except the operon *istR-1-istR-2/b4616*, which was not expressed in the samples HS60min and M-P2 h (Additional file 10). Figure 8 shows an example of a consistent operon *hemCDXY* encoding enzymes for tetrapyrrole synthesis. Although all the four genes were consistently expressed and continuously covered by the reads under different cultures and growth phases, they had similar position-dependent non-uniform read coverage along the operon, again indicating the non-uniform coverage of the libraries.

**Figure 9:**
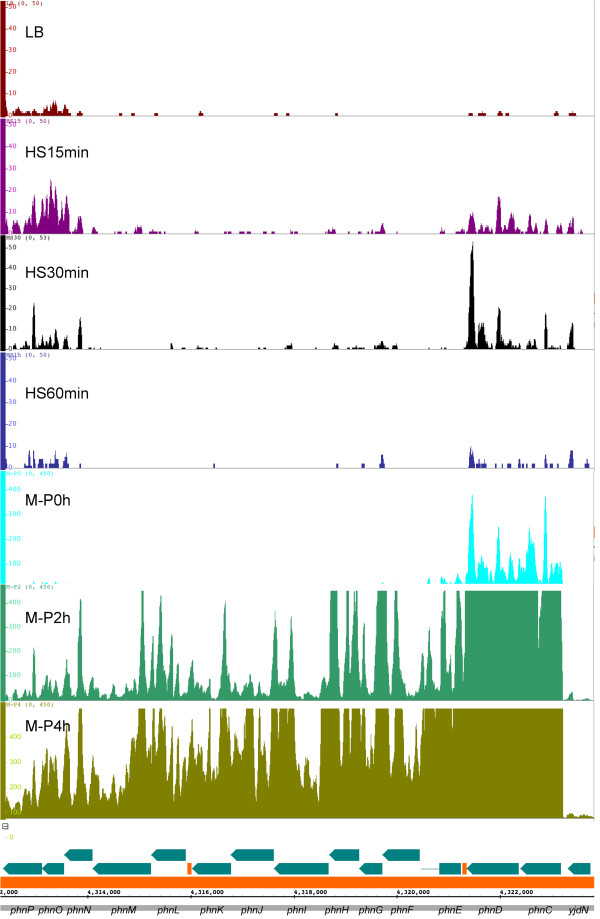
**Reads coverage of the genes in the *phn *operon.** The vertical axis is the number of reads covered at the positions. The orange and dark green bars represent the forward and reverse strands, respectively. Segments with arrows represent genes. Genes from the right to left are *yjdN, phnC, phnD, phnE, phnF, phnG, phnH, phnI, phnJ, phnK, phnL, phnM, phnN, phnO and phnP*. The graphs were generated using IGB. To make the expression levels for the 14 genes in different samples visible and comparable, the same vertical axis scale (50) is used for the LB and HS treatments, and the same vertical axis scale (450) is used for M-P treatments. Some positions with low read coverage cannot be shown while some other positions with high coverage are truncated. Note the varying levels of coverage and gaps along the operon under different cultures and growth phases, and again the similar position-dependent non-uniform coverage of the reads along the operon.

Furthermore, we consider a non-consistent operon as an alternative operon if it shares a portion of genes with another operon in other samples. As shown in Additional file 1: Table S7, from 981 (46.9%) to 1,815 (63.4%) alternative operons were detected in each sample. Thus around half of the reconstructed operons in each sample have at least one putative alternative form, a number comparable to that found in *M. Pneumonia*[23] and other prokaryotes [24,25,28,29], indicating that like many other prokaryotes [20,22-25], *E. coli* K12 seems to express enormous alternative operons under different culture conditions and growth phases, a phenomenon that is more prevalent than previously expected. An interesting example is the 14-gene operon *phnCDEFGHIJKLMNOP* coding for proteins responsible for the assimilation of C-P bond-containing phosphonates under phosphorus starvation conditions [98]. In the LB, and heat shock samples (HS15 min, HS30min and HS60 min), this operon was transcribed in several short suboperons (Additional file 1: Table S8 and Additional file 9) with low expression levels, whereas under phosphorus starvation (samples M-P2 h and M-P4 h), the *phn* genes were transcribed as a large operon *phnCDEFGHIJKLMNOP* with high expression levels (Figure 9 and Additional file 9), which is consistent with previous observations [98]. In fact, this 14-gene operon and its suboperons have been studied previously by several groups [98-101]. The *phnCDE* suboperon encoding a phosphonate transport system, was transcribed in the sample M-P0 h, and *phnF* is a repressor for this suboperon [102]. Moreover, the products of the genes *phnGHIJKLM* are essential for the C-P bond cleaving activity [103]. More recently, Jochimsen *et. al*[101] have shown that *phnGHIJK* encodes a protein complex essential for organophosphonate utilization; this suboperon was detected in the sample HS15 min. Furthermore, genes *phnNP* function as downstream processing enzymes [104], whereas the *phnO* gene is unnecessary for transport or catalysis, and may therefore have a regulatory role [103]. Finally, as shown in Figure 9, the *phnCDEFGHIJKLMNOP* operon displayed varying/decreasing expression levels along the operon, another form of the complexity of prokaryotic transcriptomes in addition to alternative operon utilization [23]. However, further investigation of this phenomenon is out of the scope of this work.

Another interesting example is the alternative utilization of the 13-gene operon *fliFGHIJKLMNOPQR* encoding proteins in the flagella of *E. coli K12* (Additional file 1: Table S9 and Additional file 9). Although the *fli* operon was expressed as a 13-gene polycistron in the sample LB, it was split into short suboperons under the treatments of heat shock or phosphorus starvation in a time dependent manner (Additional file 1: Table S9). For example, at the beginning of heat shock (the sample HS15 min), the *fli* operon was divided into four suboperons, then it was further split into six to seven suboperons (samples HS30 min and HS60 min). Interestingly, it has been shown that heat shock reduces bacterial mobility possibly through the regulatory interactions between the heat shock system and the flagellum/chemotaxis system [105]. Moreover, it has been shown that inorganic phosphorus is necessary for the motility of bacteria [106]. However, the underlying mechanisms of these observations are largely unknown. Therefore, our results might provide a possible molecular explanation of these earlier observations: the extreme conditions (heat shock/phosphorus starvation) alter the expression of flagella proteins by changing the patterns of alternative usages of the *fli* operon, thus influence the motility of the bacterial cells.

### Condition-dependent asRNA and ncRNA transcriptions appear to be prevalent in *E. coli* K12

**Figure 10:**
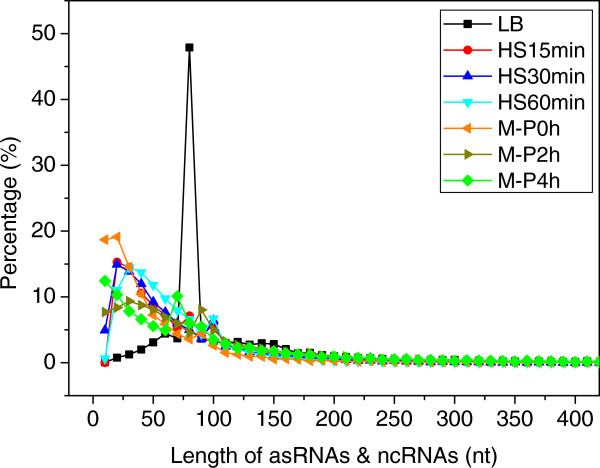
**Distribution of the length of assembled asRNA and ncRNAs.** For clarity, only the range of 1 ~ 400 nt is shown, but some asRNA can be longer than 1,000 nt.

Intriguingly, about 0.35 ~ 0.95% and 5.27 ~ 9.79% of our uniquely mapped read were mapped to the antisense strand of annotated open reading frames (ORFs) and intergenic regions, respectively (Figure 1). We consider the assembled transcripts from these reads as putative asRNAs and ncRNAs, respectively. As shown in Figure 10, majority of these putative asRNAs and ncRNAs have a length of 20 ~ 200 nt, while some can be > 1,000 nt long. Interestingly, majority (72.23 ~ 89.54%) of ORFs were predicted to have asRNA transcriptions (Additional file 1: Table S10), which is consistent with an earlier studies showing that 3,000 ~ 4,000 ORFs had asRNA transcriptions using tiling array [43]. However, a recent study [44] identified only about 1,000 asRNA in the same genome under similar growth conditions using directional RNA-seq. This discrepancy might be due to different techniques and analysis methods used. Furthermore, 1,942 ~ 2,780 (51.37 ~ 72.74%) out of the 3,808 intergenic regions had putative ncRNA transcriptions in a condition- and/or growth phase-dependent manner (Additional file 1: Table S10). To evaluate the accuracy of our assembled asRNAs and ncRNAs, we compared them with the 112 known asRNA and ncRNAs compiled by Storz’s group [55,56] and RegulonDB [53], and found that our results recovered 102 (91%) of these 112 known asRNA and ncRNAs (Additional file 11). Thus, TruHMM has also achieved rather high sensitivity in assembling asRNAs and ncRNAs. However, the authenticity and functions of the remaining putative novel asRNAs and ncRNAs need to be further investigated. The assembled putative asRNAs and ncRNAs in the seven samples are listed in Additional file 12 and Additional file 13, respectively.

### Some hypothetical genes are transcribed

Although *E. coli* K12 is probably the best studied and understood model organism, researchers have not completely defined even its coding genes. For instance, there are still 36 sequences labelled as hypothetical protein genes as of this writing in the RegulonDB [53]. Interestingly, we found that all these 36 hypothetical genes were transcribed in at least one of our seven samples (Additional file 14), and 21 (*b0050, b0137, b1356, b1382, b1419, b1446, b1457, b1607, b1952, b1998, b3471, b3638, b3937, b4325, b4335, b4336, b4593, b4596, b4610, b4615* and *b4620*) of them were expressed in all the seven samples, suggesting that they are highly likely to be true protein coding genes. Furthermore, 20 of them formed multi-gene operons with other known genes (Additional file 14). The functions of these known genes might provide hints to possible functions of the associated hypothetical genes for “guilt by association”.

## Discussion

Although a few high throughput studies have attempted to delineate the architecture of *E. coli* K12 transcriptomes [43,44,57,58], they mainly focused on identifying TSSs [57,58], promoters [58] and other features [57]. Thus we still lack a good understanding of the level of the complexity of the transcriptomes in *E. coli* K12, from which we gained most of our knowledge about transcription in bacteria, but the more recent revolutionary view of the high complexity and dynamics of prokaryotic transcriptomes. Therefore, there is an urgent need for a better understanding of the complexity of the transcriptomes in this most widely-used model Gram-negative bacterium, in particular, when the same highly complex and dynamic transcriptomes have recently been revealed in its counterpart model Gram-positive bacterium *B. Subtilis*[25]. To fill the gap, we have profiled the transcriptomes in *E. coli* K12 during the course of heat shock and phosphorus starvation conditions using a highly strand-specific RNA-seq method that can capture various forms RNA transcripts, in conjunction with a highly accurate full-length transcript assembler, TruHMM. Indeed, as has been widely reported in many other prokaryotes [24-29], we have also identified numerous putative novel and/or alternative operons and TSSs, as well as novel putative asRNAs and ncRNAs in *E. coli* K12. More importantly, the transcription patterns of these putative alternative operons, asRNAs and ncRNAs were highly dependent on the growth phases and culture conditions of the bacterium, suggesting that they might play important roles in the physiology of the bacterium. In the future, it would be very interesting to study how the alternative operons, asRNAs and ncRNAs are related to transcriptional and translational regulations and cellular functions, in particular in responses to environmental cues. Furthermore, the molecular mechanisms that lead to the highly complex and dynamic transcriptomes in *E. coli* K12 and other organisms also warrant further investigations.

Based on the ever increasing body of evidence [20-22], and the data presented in current study, it is highly likely that prokaryotes generally have highly dynamic and complex transcriptomes to cope with environmental changes. The failure to observe such highly complex and dynamic transcriptomes in some earlier studies [31-42], and the inconsistent results in *E. coli* K12 and *B. subtilis*[25,30], might well be due to the limitations of experimental and computational methods used in these studies. For instance, although an earlier study [30] did not detect alternative operon utilizations in *B. subtilis* using tiling arrays under two culture conditions, a more recent study [25] observed highly prevalent condition-dependent operon utilizations as well as numerous asRNA and ncRNA transcriptions using higher resolution tiling arrays and more sophisticated computational analysis in ~120 culture conditions. Furthermore, although Selinger *et al.*[43] found that up to 3,000 ~ 4,000 *E. coli* K12 genes had asRNA transcriptions using directional tilling arrays, Dornenburg *et al.*[44] only identified about 1,000 asRNAs in the same genome under similar growth conditions using a directional RNA-seq technique. Our results is in excellent agreement with the former results [43], as we detected that 72.23 ~ 89.54% annotated genes have putative asRNA transcriptions (Additional file 1: Table S10). Thus again asRNA transcription appears to be more prevalent than originally anticipated in *E. coli* K12. With the continuous drop in costs of the NGS technologies, directional RNA-seq becomes a routine technique to profile transcriptomes in thousands of sequenced prokaryotic genomes. We expect that highly complex and dynamic transcriptomes will be identified in more and more prokaryotes using improved directional RNA-seq techniques and analysis tools. The experimental methods and the transcripts assembler that we developed in this study can add in these efforts.

**Figure 11:**
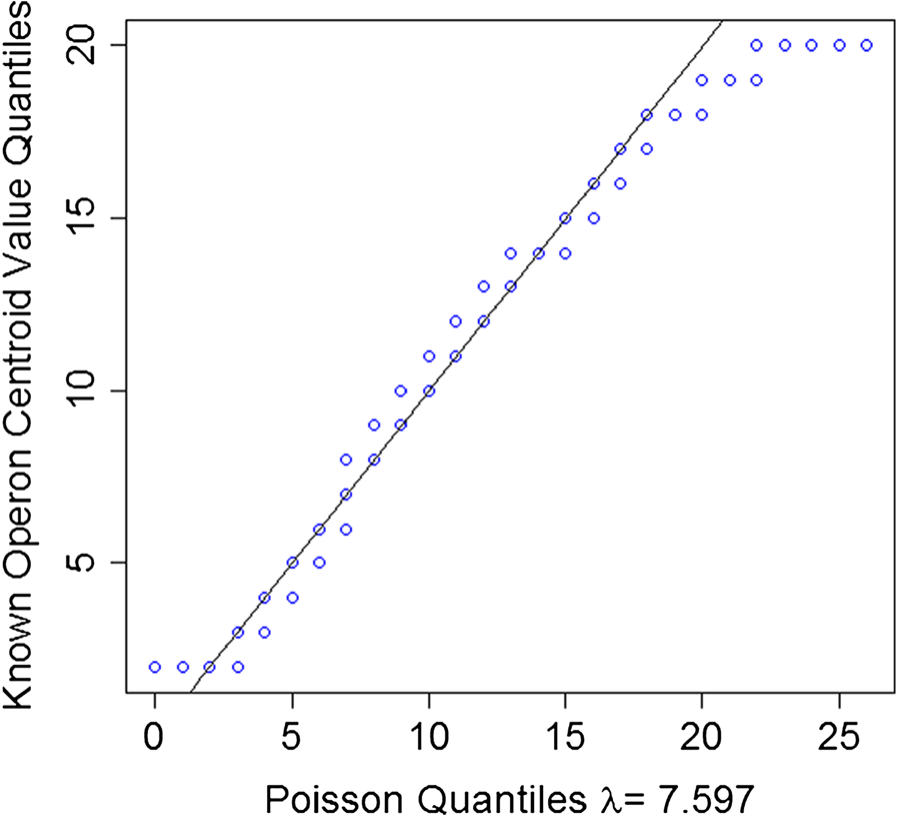
**QQ-plot comparing the distribution of centroid coverage values of the positive training set in all the samples but LB with the fitted Poisson distribution.** Deviation of a data point from the line y = x indicates its deviation from the theoretical Poisson distribution. Parameters of the Poisson distribution are estimated using the maximum likelihood method.

Specifically, our directional RNA-seq libraries preparation method based on the Illumina small RNA-seq prep method is highly strand-specific, avoiding potential genomic DNA contaminations. Our method is also capable to capture various types RNA transcripts, including mRNA and small RNAs such as asRNAs and ncRNAs, eliminating the need to prepare two libraries targeted to mRNAs and small RNAs separately [34]. Additionally, before the advent of a routine full-length RNA sequencing technology, reference-based assembly of full-length transcripts is probably the best choice and a necessary step to analyze the transcriptomes using RNA-seq short reads. Due to the highly labile nature and various technical biases introduced during the sequencing library preparation [66,72-80] and the sequencing process per se [81], transcribed regions are highly non-uniformly covered, and more seriously, a considerable portion of a transcribed region may not be covered by the reads, resulting in uncovered gaps in transcribed regions [62-67]. Our assembler TruHMM has effectively addressed these issues. TruHMM differs from an earlier HMM based method for analyzing transcriptomes in *B. anthracis*[94] in the several important aspects, and overcomes its shortcomings. First, by arbitrarily dividing read coverage values of genes into several bins, the earlier HMM [94] contains multiple expression states that are not directly connected, thus in principle it cannot assemble transcripts with highly non-uniform coverage. By contrast, TruHMM uses only a single state to model a wide range of read coverage along a transcript, thus it is able to assemble transcripts with highly non-uniform coverage. Second, the earlier method assumes a first order dependence of the mapped reads [94], which cannot effectively bridge the larger and prevalent uncovered gaps along a transcribed region as we see in our and other RNA-seq datasets. In contrast, we treat the gap problem explicitly by using a sliding-window based centroid coverage values, which as we have demonstrated in this paper, can largely relieve the gap problem. Third, the earlier method empirically assigns emission probabilities to several expression states [94]. By contrast, we derived the emission probabilities by fitting our centroid read coverage values to a Poisson distribution, which nicely models the highly non-uniform read coverage phenomenon (Figure 11). Lastly, by using a post processing strategy, our algorithm can accurately predicted TSSs, whereas the early method lacks such capability. For these reasons, our algorithm has largely solved the highly non-uniform coverage problem as well as the prevalent gap problem in assembling prokaryotic transcripts using RNA-seq short reads. Indeed, when evaluated on the seven RNA-seq datasets that we generated in *E. coli* K12 as well as datasets produced in *H. pylori*, TruHMM has achieved rather high performance in assembling operons (Figures 5 and 6, and Additional file 1: Tables S3 and S4) and locating TSSs (Figure 7, and Additional file 1: Table S6) in both our *E. coli* K12 datasets and the earlier *H. pylori* datasets. TruHmm also was able to accurately assemble asRNAs and ncRNAs as it recovered 102 (91%) of the 112 known such RNAs in *E. coli* K12 [3] (Additional file 11). Equally importantly, the performance of TruHMM also is very robust as we have demonstrated that the *E. coli*-trained parameters can be used to assemble the transcripts in *H. pylori* and vice versa, while achieving in both the cases exactly the same results as being done using the parameters trained on their own verified operons. Therefore, one can use our trained parameters to assemble transcripts in a different organism when enough known operons in the organism of interest are not available for training the parameters.

Another interesting and rather prevalent phenomenon called dynamic operon transcription is recently revealed by transcriptome profiling studies in *M. Pneumonia*[23] and *B. subtilis*[25] using high density tiling arrays that give more uniform signal coverage along genes albeit at lower resolution [23,25]. Dynamic operon transcription is characterized by varying levels of transcription along an operon, resulting in staircase like transcription levels between adjacent genes in the operon [23,25]. This phenomenon also is clearly seen in our datasets (examples are shown in Figure 9). However, TruHMM in its current form is unable to detect such dynamic operon transcription events due to the highly non-uniform read coverage along genes in an operon. Furthermore, if multiple alternative operons start at the same TSS, but terminate at different TTS in the same sample, TruHMM will fail to detect such coexisting alternative operons in the same sample. Clearly, to solve these problems, one needs to transform the highly non-uniform read coverage along the genes into a more uniform one by effectively correcting the aforementioned technical biases in the current RNA-seq methods, or relies on a better sequencing technology with minimal read bias, or capable of sequencing transcripts in their full-length. In addition, TruHMM might not be able to separate overlapping transcripts if the downstream transcript has no outstanding primary TSS. Finally, additional sequencing library targeted to the intact 5’-end of RNAs might be needed in order to identify all possible TSSs in a sample.

## Conclusions

Using a highly efficient and strand-specific RNA-seq method combined with a highly accurate and robust algorithm and tool, TruHMM for assembling full-length transcriptomes, we showed that alternative operon utilizations in *E. coli* K12 appear to be more prevalent than originally anticipated, and that a large portion of ORFs and intergenic regions of the genome appear to have asRNA and ncRNA transcriptions, respectively. Furthermore, the patterns of alternative operon, asRNA and ncRNA transcriptions are dependent on the culture conditions and growth phases of the bacterium, thus they might play important roles in the physiology of the bacterium. Furthermore, with the recognition of the highly complex nature of prokaryote transcriptomes and the wide application of RNA-seq techniques in the prokaryotes research community, TruHMM can also be very useful for biologists to reveal the complexity of transcriptomes and the underlying molecular mechanisms in all sequenced prokaryotic genomes.

## Methods

### Bacterial culture

A frozen stock of *Escherichia coli* K12 strain MG1655 (a gift from Dr. Todd Steck, Department of Biology, the University of North Carolina at Charlotte) was thawed, inoculated in LB medium in a test tube by 1:100 dilution and cultured overnight at 37°C and 250 rpm. The cells were then transferred to fresh LB medium in a flask by 1:100 dilutions, and cultured at 37°C and 250 rpm. When the cells grew to the log phase with an optical density at 610 nm [OD_610_] of 0.87, they were spun down at 3,200 g for 25 min. For heat shock treatment (HS), the cell pellets were resuspended in the same volume of MOPS medium (100 ml of 10X MOPS mixture, 880 ml of sterile H_2_O, 10 ml (0.132 M) KH_2_PO4 and 10 ml of 20% glucose, Teknova, Hollister, CA), and incubated at 48°C and 250 rpm. For phosphorus-starvation treatment (M-P), the cell pellets were resuspended in the MOPS medium without KH_2_PO4. Three milliliter cell suspension were collected in a tube containing 1.5 ml RNA Later (Invitrogen) immediately after the cell pellets were resuspended in the indicated medium (0 min) and at the indicated time points thereafter (HS:15 min, 30 min and 60 min; M-P: 0 hrs, 2 hrs, 4 hrs). Cells were spun down at 6,000 g, 8 min and -4°C, and the pellets were resuspended in 1.5 ml of *RNAlater*. The samples were stored at -80°C until use.

### Isolation and enrichment of mRNA

Total RNA was isolated using a RiboPure™ -Bacteria Kit (Ambion) following the manufacturer’s instructions. Once isolated, ~10 μg total RNA was treated with 8 units DNase (Invitrogen) twice to remove genomic DNA, and the complete removal of DNA was confirmed bythe absence of the product of 35 cycles PCR amplification of a 196 bp fragment of the *crp* gene (5’-primer: AGCATATTTCGGCAATCCAG; 3’-primer: TACAGCGTTTCCGCTTTTTC). To enrich mRNAs and other transcripts, majority of rRNAs were removed from the DNase-treated total RNA using a MICROBExpress kit (Ambion) following the manufacturer’s instructions.

### Construction of directional RNA-seq libraries

In our early stage of experiments, sequencing was done on an Illumina GAII platform at the sequencing core facility of the University of North Carolina at Chapel Hill, and the directional RNA-seq libraries were constructed by following an Illumina’s instruction using their Small RNA Sample Prep Kit with some modifications. Briefly, after the purified mRNA was fragmented using a RNA fragmentation kit (Ambion), the fragmented RNA was treated with Antarctic phosphatase (NEB) to remove the 5’-tri-phosphate groups of RNAs with an intact 5’-end. A mono-phosphate group was then added back to the 5’-end of fragmented RNAs by polynucleotide kinase (PNK, NEB) in the presence of 10 mM ATP. The v1.5 sRNA 3’ Adaptor (5’/5rApp/ATCTCGTATGCCGTCTTCTGCTTG/3ddC/) was ligated to the 3’-end of fragmented RNAs using truncated T4 ligase 2 (NEB), and the SRA 5’ RNA adaptor (5’GUUCAGAGUUCUACAGUCCGACGAUC) was ligated to the 5’-end of fragmented RNAs using T4 ligase. To preserve short inserts from small RNAs we omitted the size selection step after PCR application of inserts. In our later experiments, sequencing was done on an Illumina HiSeq 2000 platform at David H. Murdock Research Institute of the North Carolina Research Campus (Kannapolis, NC), and we constructed the directional RNA-seq libraries using Illumina’s TruSeq Small RNA Sample Prep Kit, so that multiplex sequencing can be achieved by using the barcoded PCR primers. The details of the method will be described elsewhere (Dong, Li and Su). Briefly, after similar treatments as described above, the 5’ Adapter (RA5: 5’ GUUCAGAGUUCUACAGUCCGACGAUC), and 3’ Adapter (RA3: 5’ TGGAATTCTCGGGTGCCAAGG) were ligated to 5’- and 3’-end of fragmented RNAs, respectively. Reverse transcription-PCR (RT-PCR) was performed using SuperScript II Reverse Transcriptase Kit using the SRA RT primer, followed by 16 cycles of PCR amplification. Again, the size selection was omitted on PCR products to preserve short inserts from possible small RNAs. Single-end sequencing on the Illumina GA II platform was done with 76 cycles, while that on the HiSeq 2000 platform was done with 100 cycles. Some samples (HS15 min and M-P4 h) were sequenced on both platforms.

### Mapping and filtering RNA-seq reads

The genome sequence and annotation files of *E. coli* K12 substr. MG1655 were obtained from NCBI (http://ftp.ncbi.nlm.nih.gov/genomes/Bacteria/Escherichia_coli_K_12_substr__MG1655_uid57779/), and the experimentally verified operons in the bacterium were downloaded from RegulonDB [53] (http://regulondb.ccg.unam.mx/). Additional 112 experimentally verified small RNAs in *E. coli* were obtained from Storz’s group (http://cbmp.nichd.nih.gov/segr/ecoli_rnas.html). A total of 4,501 annotated genes (also including pseudo genes and small RNAs) are included in this analysis. As the reads were not size-selected during the library construction, we trimmed the 3’ adapters attached to some short insertions. Adapter-free reads with lengths of <10 nt were discarded; the remaining reads were mapped to the *E. coli* K12 genome using Bowtie [87]. For the reads of length 10–14, 15–29 and ≥30 nt, up to 1, 2, and 3 mismatches were allowed, respectively. Since over 99.6% of the multiple mapped reads in each sample were from duplicated tRNA/rRNA genes (data not shown), only uniquely mapped reads were used for further analysis. The alignment of mapped reads to the reference genome was visualized by Integrated Genome Browser (IGB) [107]. To map the directional RNA-seq reads of *H. pylori*[24], we trimmed the polyA tails of the original datasets, which were introduced during the library preparation, and mapped the reads to the reference genome using Bowtie with the same parameter settings as for *E. coli* K12.

### Normalization of the mapped counts

**Figure 12:**
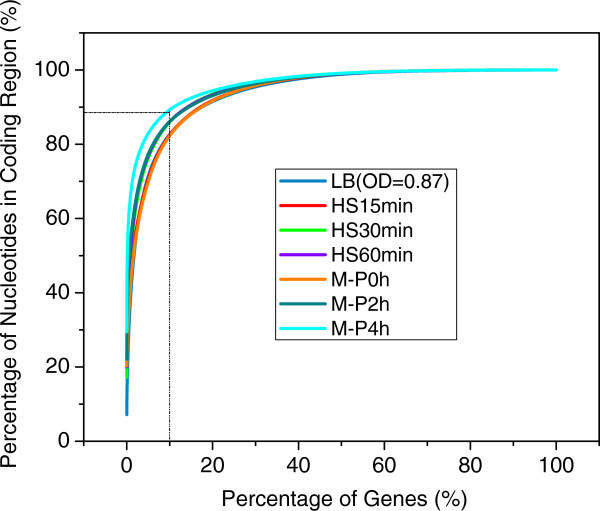
**Impact of highly expressed genes on the mapped nucleotides in coding regions.** Genes were sorted in the descending order of their number of mapped nucleotides in reads. The top 10 percent of genes with the highest read counts contribute to around 80% ~90% mapped nucleotides in the coding regions.

Normalization of the mapped read counts is crucial for differential expression detection using RNA-seq [108], as different samples may have different total read counts, i.e. sequencing depths, as well as various biases mentioned earlier. The most commonly used normalization methods include reads per kilobase of exon model (or ORF) per million mapped reads (RPKM) [62], fragments per kilobase of transcript per million fragments mapped reads (FPKM) [71], the hypergeometric model [109] and other more recent sophisticated model-based methods [63,64,66,67,77,78,110,111]. However, it has been shown that these global normalization methods are strongly affected by a small proportion of highly expressed genes in the published datasets, leading to biased estimation of gene expression levels across different conditions [108]. As shown in Figure 12, our datasets are no exception to the problem as around 10% of genes with the highest number of mapped nucleotides contribute up to 80% ~ 90% of mapped nucleotides in the gene-coding regions across all the seven samples. Inspired by [108] and also for computational efficiency, in this study we used N* defined as the total nucleotide counts minus the counts of the top 10% of genes with the highest counts to scale the gene expression levels in each sample, instead of using the total counts of mapped nucleotides in each sample.

Furthermore, because our mapped reads have different lengths (see Results), instead of using the mapped read counts per gene, we used the mapped nucleotide counts per gene to measure the gene expression levels defined as “Nucleotides Per Kilo base of transcript per Billion nucleotides mapped” (NPKB):

(1) $$\text{NPKB}=\frac{n}{\frac{N^{*}}{10^{9}}\times \frac{L}{10^{3}}},$$

Where *n* is the number of nucleotides of the reads mapped to the transcript, N* our normalization factor defined above, and *L* the length of the transcript. Clearly, when all reads have the same length, NPKB and RPKM differ by a constant scaling factor. A similar method has been used earlier [60], except that our NPKB is further normalized by the global scaling factor N* in each sample.

### Training the HMM

**Figure 13:**
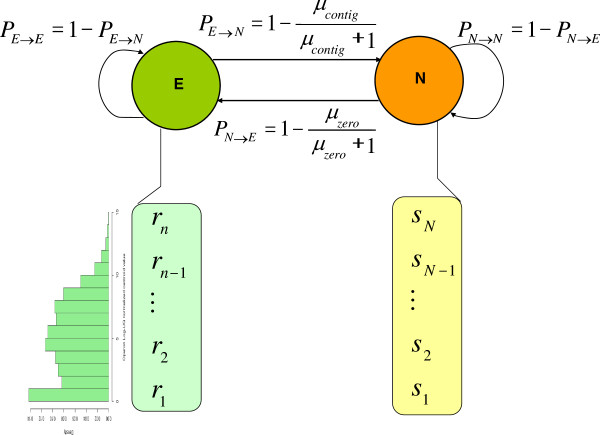
**Structure of the HMM for assembling operons/transcripts using RNA-seq reads.** E represents the expression state and N the non-expression state, Letters *r*_*1*_*, r*_*2*_*,…,r*_*n*_ are the emission values of *E*, *μ*_*contig*_ is the mean length of sufficiently expressed contigs in the positive training set; and *s*_*1*_*, s*_*2*_*,…, s*_*N*_ are the emission values of *N*, and *μ*_*zero*_ is the mean length of the non-expressed regions in the negative training set.

An HMM is a machine-learning algorithm that can be used to decode the path of hidden states that generate a sequence. In this paper, we use an HMM to infer whether or not a segment of a strand of DNA is consecutively transcribed given the expression values obtained from the mapped reads. The model consists of two states: the expression state *E* and non-expression state *N* (Figure 13).

#### ***Selection of expressed adjacent operon pairs***

**Figure 14:**
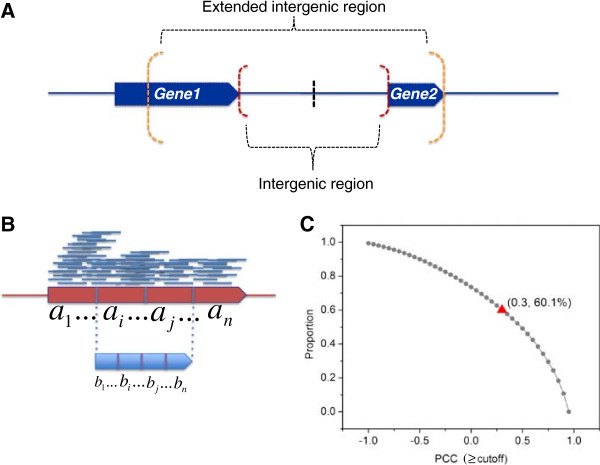
**Selection of known adjacent operon pairs for training and evaluation. A**: The intergenic region between two adjacent genes in an operon is doubled by extending its two ends in the two flanking genes. **B**: A sufficiently expressed gene is equally divided into *n* bins, and its central half is further equally divided into *n *bins. The NPKB values for each bin of a gene and of its central portion are *a*_*1*_,…,*a*_*i*_*,…, a*_*j*_*, …, a*_*n*_ and *b*_*1*_*,…, b*_*i*_*,…, b*_*j*_*,…, b*_*n*_*, *respectively. An extended intergenic region is similarly divided by treating it as a “gene” with the intergenic region being the central portion of the “gene”. **C**: Distribution of PCC values between the two vectors for sufficiently expressed genes with a bin size *n* = 4. We choose 0.3 as the cutoff of PCC value since 60.1% of sufficiently expressed genes can be included.

A gene was considered to be sufficiently expressed if over 50% of its length was covered by at least one read and at least 20 nt of both of its termini were covered by at least one read. We used the 476 experimentally verified operons in RegulonDB (Additional file 2) to train the parameters of the HMM and to evaluate the performance of our algorithm. Since these operons were not necessarily expressed in our samples, and alternative operon utilizations could be very prevalent, as the first step to construct a positive operon set in a sample, we selected a pair of adjacent genes in a known operon (adjacent operon pair) if they met the following two criteria: 1) both genes were sufficiently expressed and over 50% of the length of their intergenic region were covered by at least one read in the sample; and 2) the correlation between the expression levels of the two genes and their intergenic region was greater than a cutoff. To compute the correlation between the expression levels of the two genes and their intergenic region, we extended the two ends of the intergenic region into the two flanking genes to double its length or extended until the other end of either gene was reached (Figure 14A). We equally divided the extended intergenic region as well as the intergenic region into *n* bins, and thus the expression levels (NPKB) over these bins formed two *n*-element vectors (Figure 14B). Pearson correlation coefficient (PCC) between the two vectors was used to quantify the correlation between the expression levels of the two genes and their intergenic region. To find an appropriate cutoff, we similarly divided a sufficiently expressed gene as well as its central half into *n* equal bins, and computed the correlation of the expression levels between the whole gene and its central half. We reason that for an expressed adjacent operon pair, the PCC value between the intergenic region and the extended intergenic region should follow the same distribution of the PCC value between the central half of an expressed gene and the whole gene, since an adjacent operon pair and their intergenic region should be expressed in a similar way as the different parts of a gene. The distribution of the PCC value between the central half and the whole gene (*n* = 4) is shown in Figure 14C. We chose 0.3 as the cutoff for our second criterion to select positive adjacent operon pair since this would allow us to include over 60% of sufficiently expressed genes.

#### ***Positive and negative training sets***

To train the HMM, we constructed a positive training set in a sample by simply stitching the known adjacent operon pairs that met the two criteria described above to form a large operon if they are parts of a known operon according to RegulonDB. These positive training sets in the seven samples are listed in Additional file 2. To construct a relatively large negative training set in a sample, we included all the uncovered regions in the genome excluding the ones inside the sufficiently expressed genes in the sample.

#### ***Positive and negative testing sets***

We evaluated the operon prediction accuracy using two methods: one was based on adjacent operon pairs, and the other on the entire operon structure using all the gene pairs of a known operon. For the first method, we constructed a positive testing set in a sample, consisting of sufficiently expressed adjacent operon pairs, and a negative testing set consisting of known adjacent non-operon pairs that were both sufficiently expressed in the sample. A known adjacent non-operon pair consisted of either the first gene of a known operon and its immediate upstream gene, or the last gene in a known operon and its immediate downstream gene, as long as the intergenic region of the gene pair had at least one uncovered region, regardless of its length. For the second method, we constructed a positive testing set in a sample, consisting of all pair-wise combinations of the genes in a sufficiently expressed operon, and a negative testing set consisting of the gene pairs between the genes of the operon and the immediate upstream or immediate downstream gene, given that the known adjacent non-operon pairs had no overlapping un-translated region (UTR) and that all these relevant genes were sufficiently expressed.

#### ***Leave-one-out cross validation***

We employed a leave-one-out cross validation strategy to evaluate the performance of our algorithm. Specifically, we used the positive training sets and negative training sets in (*n*-1) samples to train the emission and transition probabilities of the HMM, and used the positive testing set and the negative testing set in the remaining sample to test the trained model.

#### ***Training emission probabilities***

The number of reads mapped to a specific position (nucleotide) in the reference genome is denoted as “coverage” of the position in this paper. To deal with the uncovered gap problem, we used a sliding window to compute the centroid coverage of each position on a strand of DNA, assuming that if the flanking regions of a position are transcribed, it is very likely that the position itself also is transcribed. Specifically, given a window size *L* (*L* is an odd number), the centroid coverage of the nucleotide *i* in the middle of the window is defined as:

(2) $$\text{Centroid}\left(i\right)=\text{log}\left(\frac{10^{9}}{N^{*}}\left(\frac{1}{L}\sum_{k=i-\left(L-1\right)/2}^{i+\left(L-1\right)/2}\text{Coverage}\left(k\right)+1\right)\right),$$

Where *i* is the *i-th* position (nucleotide) on the chromosome. N* the normalization factor defined in equation (1), *L* the window size, and Coverage (*k*) the coverage of position *k* on the genome. Note that a pseudo count of 1 is added to the coverage value of each window. The optimal window size is determined by balancing two goals with opposite effects: to cover as many gaps as possible and to exclude as many interoperonic regions as possible. See Results for the details of window size selection.

The emission signals of the states *E* (*r*_*1*_*, r*_*2*_*,* …,) and *N* (*s*_*1*_*, s*_*2*_, …) are the centroid coverage values of the nucleotides in the reference genome. We used the positive training sets to estimate the emission probabilities of the signals of *E*. The distribution of centroid coverage values of the positive training set from all samples except LB is shown in Figure 11. The QQ plot indicates that the centroid coverage values of the positive training set approximately follow a Poisson distribution, which is consistent with the earlier results [108]. Thus, the emission probability of the centroid coverage values in the state *E* could be computed by the Poisson distribution, whose parameters were estimated with the maximum likelihood method. Since our negative training set were virtually not covered by reads, the signals that the state *N* emits should be the centroid coverage values with zero coverage,

(3) $$\text{log}(\frac{10^{9}}{N^{*}}(\frac{1}{L}\sum_{k=i-\left(L-1\right)/2}^{i+\left(L-1\right)/2}0+1)).$$

We arbitrarily assigned a high probability 1-10^-20^ for N to emit this value, and a low probability10^-20^ for *N* to emit any other values. The value 10^-20^ is also a pseudo probability to avoid zero probability for decoding the HMM later.

#### ***Training transition probabilities***

We chose to model the lengths of both expressed and non-expressed regions with geometric distributions, though other distributions may provide a fit. To this end, let *P*_*ij*_ be the transition probability from state *i* to *j*. To estimate the transition probabilities *P*_*EE*_ and *P*_*EN*_, i.e., the probability to stay in the state *E* and to transit from the state *E* to the state *N*, respectively, let *X* be the length of a consecutively expressed region of the genome. Under the Markov assumption, *X* should follow a geometric distribution,

(4) $$P\left(X=n\right)=P_{\mathrm{EE}}^{n}\cdot \left(1-P_{\mathrm{EE}}\right)$$

Similarly, let *Y* be the length of a consecutively non-expressed (uncovered) region of genome, then *Y* also follows a geometric distribution,

(5) $$P\left(Y=n\right)=P_{\mathrm{NN}}^{n}\cdot \left(1-P_{\mathrm{NN}}\right).$$

**Figure 15:**
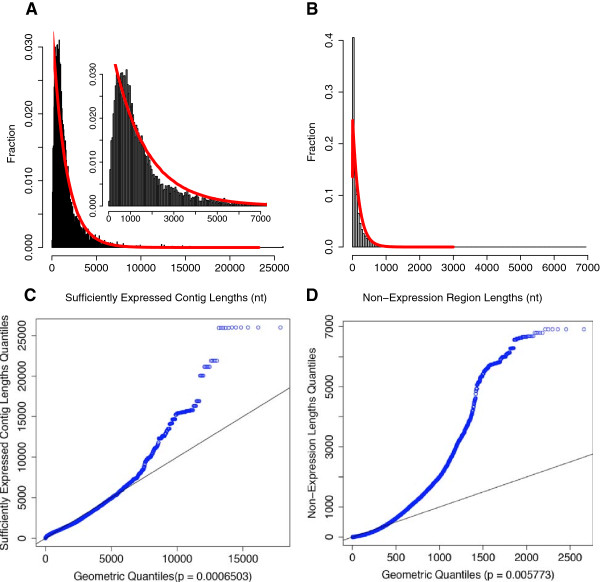
**Distributions of the lengths of sufficiently expressed contigs and non-expressed regions in all the samples except LB. A**: Histogram of the lengths of sufficiently expressed contigs (bin size =50 nt). The curve is the geometric distribution with the success probability *p* = 0.0006503 estimated by the maximum likelihood method. The inset is a blow-up view of the region of length 1 ~ 7,000 nt. **B**: Histogram of the lengths of non-expressed regions (bin size =50 nt). The curve is the geometric distribution with p = 0.00577 estimated by the maximum likelihood method. **C**: QQ-plot of the lengths of the sufficiently expressed contigs against the fitted geometric distribution. **D**: QQ-plot for the lengths of non-expressed regions against the fitted geometric distribution.

To generate “full length transcripts training sets”, we simply stitched overlapping reads along the body of a known gene or operon to assembly larger contigs. We consider as sufficiently expressed contigs those that cover at least 50% of a known gene or an adjacent operon pair of a known operon. We used the lengths of such contigs to estimate the probability of staying in the state *E* as *P*_*EE*_ = *E*(*X*)/(*E*(*X*) + 1), where *E*(*X*) is the mean length of sufficiently expressed regions. *E*(*X*) can be determined from the sufficiently expressed contigs in the samples. For example, using such contigs from all the samples except LB, we obtained *E*(*X*) = 1,537 nt and *P*_*EN*_ = 0.0006503 (Figure 15A). Notably, the vast majority of contigs have a length shorter than 8,000 nt. Furthermore, we used the lengths of non-expressed regions in the negative training sets to estimate the probability of remaining in the state *N* as *P*_*NN*_ = *E*(*Y*)/(*E*(*Y*) + 1), where *E*(*Y*) also can be determined from raw coverage data, for example, *E*(*Y*) = 127 nt, and *P*_*NN*_ = 0.005773 for all the negative training sets from all samples except LB (Figure 15B). The derivation of transition probabilities estimations is given in Additional file 1: Figure S9. The QQ plot indicates that although not precisely, the lengths of the sufficiently expressed contigs can be largely modelled as a geometric distribution (Figures 15A and C), in particular when the length of contigs is shorter than 7,000 nt. However, the lengths of non-expression regions could not be modelled by a geometric distribution (Figures 15B, D), probably because of the uncovered gaps in the expressed regions, which were much shorter than authentic non-expressed regions. Nevertheless, we found that this deviation had little effects on the performance of the algorithm (see Results). We should point out that although several previous studies have shown that the lengths of exons in eukaryotes or ORFs in prokaryotes do not follow a geometric distribution [112,113], and we have confirmed this in *E. coli* K12 (Additional file 1: Figures S10A and C), it is not very surprising that the lengths of prokaryotic mRNA transcripts largely follow a geometric distribution (Figures 15A and C). This result might be due to the fact that the length of a prokaryotic mRNA transcript is not limited by the lengths of its constituent ORFs, rather, it also depends on the lengths of the 5’ UTR, constituent intergenic regions and 3’ UTRs. The lengths of the UTR regions are known to follow geometric distributions, at least in eukaryotes [112,114]. In addition, the lengths of all of the intergenic sequences are known to follow a geometric distribution [112] (Additional file 1: Figures S10B and D). Therefore the lengths of prokaryotic mRNA transcripts behave very differently from those of ORFs.

### Reconstruction of full length transcripts/operons

We used the Viterbi algorithm [115] to decode the path of the states that best explains the centroid coverage values of a region of DNA. If a string of adjacent genes are connected by a consecutive sequence of expressed states, then these genes are predicted to form an operon. Furthermore, we stitched two candidate adjacent operons, for instance, A-B and B-C, to obtain the full length transcripts/operons A-B-C. If over half of the length of a terminal gene is predicted to be expressed, this gene is considered as a member of the predicted operon, otherwise the expressed part of the terminal gene is only considered as the UTR of the operon. The TSS and TTS of an assembled operon/transcript were determined by the locations of its 5’-end and the 3’-end, respectively.

However, errors could be introduced in the assembled operon/transcripts, and thus need to be fixed. Specifically, due to the short length of the reads, if a sub-operon/transcript overlaps with an upstream operon/transcript that are expressed in a sample, the algorithm will assemble the two operons/transcripts into a single one, missing the downstream sub-operon/transcript. Furthermore, if multiple alternative operons with different TSSs are transcribed in a sample, the assembled transcripts will be the possible longest alternative operon used in the sample. To identify such possible alternative operons as well as their TSSs, we applied to each assembled operon/transcript the following procedure based on the observation that there is often an abrupt increase in the read coverage at a TSS. The procedure attempts to identify a possible TSS inside an assembled transcript by detecting the position at which an abrupt increase in the coverage occurs in the upstream region of a transcribed gene. Specifically, for each assembled operon/transcript with a long 5’ UTRs (>50 nt), we used two sliding windows of size 2*w*_1_ and 2*w*_2_ around the position *i*, [*i-w*_1_, *i + w*_1_] and [*i-w*_2_, *i + w*_2_], *w*_1_ > *w*_2_ > 0, to scan each position of the 5’ UTR associated with the first gene in the operon, and compute coverage ratios *r*_1_(*i*) and *r*_2_(*i*) between the downstream and upstream half windows, defined as follows,

(6) $$\gamma_{1}\left(i\right)=\left\{\begin{array}{l} \left(\sum_{k=i+1}^{i+w_{1}}(\text{Coverage}\left(k\right)+1\right))/\\ (\sum_{k=i-w_{1}}^{i-1}\left(\text{Coverage}\left(k\right)+1\right))\begin{array}{cc} & \text{forwardstrand} \end{array}\\ \left(\sum_{k=i-w_{1}}^{i-1}(\text{Coverage}\left(k\right)+1\right))/\\ (\sum_{k=i+1}^{i+w_{1}}\left(\text{Coverage}\left(k\right)+1\right))\begin{array}{cc} & \text{reversestrand} \end{array} \end{array}\right)$$

(7) $$\gamma_{2}\left(i\right)=\left\{\begin{array}{l} \left(\sum_{k=i+1}^{i+w_{2}}(\text{Coverage}\left(k\right)+1\right))/\\ (\sum_{k=i-w_{2}}^{i-1}\left(\text{Coverage}\left(k\right)+1\right))\begin{array}{cc} & \text{forwardstrand} \end{array}\\ \left(\sum_{k=i-w_{2}}^{i-1}(\text{Coverage}\left(k\right)+1\right))/\\ (\sum_{k=i+1}^{i+w_{2}}\left(\text{Coverage}\left(k\right)+1\right))\begin{array}{cc} & \text{reverse} \end{array}\text{strand} \end{array}\right)$$

Since there must be a TSS associated with the first gene of an assembled transcript, we predict position *j* in the 5’ UTR, with the largest sum of ratios *γ*_1_(*j*) + *γ*_2_(*j*) as the TSS associated with the first gene in the assembled transcripts, i.e.,

(8) $$j=\underset{i}{\text{ArgMax}}\left[\gamma_{1}\left(i\right)+\gamma_{2}\left(i\right)\right].$$

To identify potential alternative TSSs for the downstream genes of the assembled transcripts, we used a rather strict threshold of 5-fold for the ratio *γ*_1_(*j*), to guarantee that there is an outstanding ‘jump’ of read coverage in the downstream of position *j*. In both cases, we set *w*_1_ = 80 nt and *w*_2_ = 10 nt. The TTSs were simply determined by the locations of the 3’-end of the assembled operons/transcripts.

The algorithm was encoded in C++ and perl. The software package is open-source, and can be downloaded from http://bioinfolab.uncc.edu/TruHmm_package/. We provide users the option to train their model if enough known operons are available in their genomes of interest. Otherwise users can apply our algorithm using the default settings without the need of any training.

### Motif detection in promoters

We applied MEME [96] to search for σ^70^ binding sites (Pribnow box) within 25 nt upstream of 1,742 experimentally verified TSSs. The motif profile was then used to scan for the potential Pribnow box within the [-100 nt, 100 nt] interval around the predicted TSS by the scoring function (formula (9,10,11)) we developed before [116,117]:

(9) $$S_{M}\left(t\right)=\text{max}\sum_{i=1}^{L}I_{i}\text{log}\frac{p\left(i,h\left(i\right)\right)}{q\left(h\left(i\right)\right)}$$

(10) $$I_{i}=\left(\sum_{b\in \left\{A,C,G,T\right\}}p\left(i,b\right)\text{log}\frac{p\left(i,b\right)}{q\left(b\right)}\right)/a$$

$$\begin{array}{ll} a= & \frac{n+1}{n+4}\text{log}\left(n+1\right)-\text{log}(n+4)\\ & -\frac{1}{n+4}\sum_{b\in \left\{A,C,G,T\right\}}\text{log}q\left(b\right)\\ & -\frac{n}{n+4}\text{log}\underset{b\in \left\{A,C,G,T\right\}}{\text{min}}q\left(b\right) \end{array}$$

To estimate the statistical significance of motif scores, we used a 3^rd^-order Markov model to generate 50,000 random sequences based on the transition probabilities learned from the set of experimentally verified promoters in *E. coli* K12. The distribution of the motif scores in the random sequences was used to define an empirical p-value.

### Performance metrics

To evaluate the performances of our algorithm, we use the following metrics.

Sensitivity=Recall=TPR=TPTP+FNSpecificity=1-FPR=TNFP+TNAccuracy=TP+TNTP+FP+TN+FNPrecision=TPTP+FPF-factor=2×Recall×PrecisionRecall+Precision

Where, TP (true positive) = Number of known operon pairs accurately classified as operon pairs by the model.

FP (False Positive) = Number of non-operon pairs falsely classified as operon pairs by the model.

FN (False Negative) = Number of known operon pairs falsely classified as non-operon pairs by the model.

TN (True Negative) = Number of non-operon pairs accurately classified as non-operon pairs by the model.

Sensitivity, i.e. TPR (True Positive Rate or recall) is the proportion of known operon pairs that can be correctly identified as operon pairs by the model. Specificity, i.e. 1-FPR (False Positive Rate) is the proportion of non-operon pairs that are correctly classified as non-operon pairs. Accuracy combines the two metrics to quantify the overall performance of the model. A high Accuracy value represents a low total error rate. Precision denotes the proportion of predicted positives that are true positives. F-factor combines Recall and Precision and normalized them to an idealized value.

## Abbreviations

HMM: Hidden Markov model; TruHMM: TRancription unit assembly by a Hidden Markov model; E. coli: *Escherichia coli* K12 substr MG1655 uid57779; TF: Transcription factor; H. pylori: *Helicobacter pylori* 26695 uid57787; B. subtilis: *Bacillus subtilis*; M. pneumonia: *Mycoplasma pneumonia*; UTR: Untranslated region; TSS: Transcription starting site; TTS: Transcription terminating site; NPKB: Nucleotides per kilo base of transcript per billion nucleotides mapped; TP: True positive; TPR: True positive rate; FP: False positive; FPR: False positive rate; asRNA: Antisense RNA; ncRNA: Non-coding RNA; ORF: Open reading frames; NGS: Next generation sequencing.

## Competing interests

The author declares that they have no competing interests.

## Authors’ contributions

SL designed the algorithms, and conducted analyses. XD generated the experimental data. ZS conceived the project. SL and ZS wrote the manuscript. All authors read and approved the final manuscript.

## Supplementary Material

Additional file 1 **Supporting figures and tables. Figure S1-S10 and Table S1-S10.** Click here for file

Additional file 2 **The known operons of *E. coli *K12 and training set in each sample.** Click here for file

Additional file 3 **The confirmed operons and training set in the *H. pylori *samples.** Click here for file

Additional file 4 **The confirmed TSS predictions in the *H. pylori *samples.** Click here for file

Additional file 5 **The confirmed TSSs prediction in the *E. coli *samples.** Click here for file

Additional file 6 The predicted TSSs that is not confirmed or annotated in RegulonDB. Click here for file

Additional file 7 **The potential Pribnow boxes detected in the interval [-100 nt, 100 nt] centred by the predicted TSSs with a p-value** ≤**0.05 in *E. coli.*** Click here for file

Additional file 8 **The TSSs detected and not detected by TruHMM in the *H. pylori *samples.** Click here for file

Additional file 9 **The reconstructed operons with alternative TSSs in the *E. coli *samples.** Click here for file

Additional file 10 **The consistent longest possible alternative operons across all the *E. coli *samples.** Click here for file

Additional file 11 **The combined known small RNAs from Storz’s group **[55,56]** and RegulonDB **[53]** and those we reconstructed in *E. coli *samples.** Click here for file

Additional file 12 **The predicted antisense RNAs in the *E. coli *samples.** Click here for file

Additional file 13 **The predicted non-coding RNAs in the *E. coli *samples.** Click here for file

Additional file 14 **The hypothetical proteins expressed in the *E. coli *samples.** Click here for file

## References

[B1] Liu JM Camilli A A broadening world of bacterial small RNAs Curr Opin Microbiol 2010 13 18 23 10.1016/j.mib.2009.11.004 20022798 PMC2822007

[B2] Repoila F Darfeuille F Small regulatory non-coding RNAs in bacteria: physiology and mechanistic aspects Biol Cell 2009 101 117 131 10.1042/BC20070137 19076068

[B3] Thomason MK Storz G Bacterial antisense RNAs: how many are there, and what are they doing? Annu Rev Genet 2010 44 167 188 10.1146/annurev-genet-102209-163523 20707673 PMC3030471

[B4] Georg J Hess WR cis-antisense RNA, another level of gene regulation in bacteria Microbiol Mol Biol Rev 2011 75 286 300 10.1128/MMBR.00032-10 21646430 PMC3122628

[B5] Keseler IM Collado-Vides J Santos-Zavaleta A Peralta-Gil M Gama-Castro S Muniz-Rascado L Bonavides-Martinez C Paley S Krummenacker M Altman T EcoCyc: a comprehensive database of Escherichia coli biology Nucleic Acids Res 2011 39 D583 590 10.1093/nar/gkq1143 21097882 PMC3013716

[B6] Sierro N Makita Y de Hoon M Nakai K DBTBS: a database of transcriptional regulation in Bacillus subtilis containing upstream intergenic conservation information Nucleic Acids Res 2007 36 D93 96 10.1093/nar/gkm910 17962296 PMC2247474

[B7] Chen X Su Z Xu Y Jiang T Computational prediction of Operons in *synechococcus sp.* WH8102 Genome Inform Ser Workshop Genome Inform 2004 15 211 222 15706507

[B8] Westover BP Buhler JD Sonnenburg JL Gordon JI Operon prediction without a training set Bioinformatics 2005 21 880 888 10.1093/bioinformatics/bti123 15539453

[B9] Price MN Huang KH Alm EJ Arkin AP A novel method for accurate operon predictions in all sequenced prokaryotes Nucleic Acids Res 2005 33 880 892 10.1093/nar/gki232 15701760 PMC549399

[B10] Dam P Olman V Harris K Su Z Xu Y Operon prediction using both genome-specific and general genomic information Nucleic Acids Res 2007 35 288 298 17170009 10.1093/nar/gkl1018 PMC1802555

[B11] Tran TT Dam P Su Z Poole FL 2nd Adams MW Zhou GT Xu Y Operon prediction in Pyrococcus furiosus Nucleic Acids Res 2007 35 11 20 17148478 10.1093/nar/gkl974 PMC1761436

[B12] Bergman NH Passalacqua KD Hanna PC Qin ZS Operon prediction for sequenced bacterial genomes without experimental information Appl Environ Microbiol 2007 73 846 854 10.1128/AEM.01686-06 17122389 PMC1800777

[B13] Mao F Dam P Chou J Olman V Xu Y DOOR: a database for prokaryotic operons Nucleic Acids Res 2009 37 D459 463 10.1093/nar/gkn757 18988623 PMC2686520

[B14] Taboada B Verde C Merino E High accuracy operon prediction method based on STRING database scores Nucleic Acids Res 2010 38 e130 10.1093/nar/gkq254 20385580 PMC2896540

[B15] Livny J Efficient annotation of bacterial genomes for small, noncoding RNAs using the integrative computational tool sRNAPredict2 Methods Mol Biol 2007 395 475 488 10.1007/978-1-59745-514-5_30 17993693

[B16] Tjaden B Prediction of small, noncoding RNAs in bacteria using heterogeneous data J Math Biol 2008 56 183 200 17354017 10.1007/s00285-007-0079-5

[B17] Pichon C Felden B Small RNA gene identification and mRNA target predictions in bacteria Bioinformatics 2008 24 2807 2813 10.1093/bioinformatics/btn560 18974076

[B18] Luban S Kihara D Comparative genomics of small RNAs in bacterial genomes OMICS 2007 11 58 73 10.1089/omi.2006.0005 17411396

[B19] Brouwer RW Kuipers OP Hijum SA The relative value of operon predictions Brief Bioinform 2008 9 367 375 10.1093/bib/bbn019 18420711

[B20] Toledo-Arana A Solano C Deciphering the physiological blueprint of a bacterial cell: revelations of unanticipated complexity in transcriptome and proteome Bioessays 2010 32 461 467 10.1002/bies.201000020 20486131

[B21] Sorek R Cossart P Prokaryotic transcriptomics: a new view on regulation, physiology and pathogenicity Nat Rev Genet 2010 11 9 16 19935729 10.1038/nrg2695

[B22] Filiatrault MJ Progress in prokaryotic transcriptomics Curr Opin Microbiol 2011 14 579 586 10.1016/j.mib.2011.07.023 21839669

[B23] Guell M van Noort V Yus E Chen WH Leigh-Bell J Michalodimitrakis K Yamada T Arumugam M Doerks T Kuhner S Transcriptome complexity in a genome-reduced bacterium Science 2009 326 1268 1271 10.1126/science.1176951 19965477

[B24] Sharma CM Hoffmann S Darfeuille F Reignier J Findeiss S Sittka A Chabas S Reiche K Hackermuller J Reinhardt R The primary transcriptome of the major human pathogen Helicobacter pylori Nature 2010 464 250 255 10.1038/nature08756 20164839

[B25] Nicolas P Mader U Dervyn E Rochat T Leduc A Pigeonneau N Bidnenko E Marchadier E Hoebeke M Aymerich S Condition-dependent transcriptome reveals high-level regulatory architecture in Bacillus subtilis Science 2012 335 1103 1106 10.1126/science.1206848 22383849

[B26] Passalacqua KD Varadarajan A Ondov BD Okou DT Zwick ME Bergman NH Structure and complexity of a bacterial transcriptome J Bacteriol 2009 191 3203 3211 10.1128/JB.00122-09 19304856 PMC2687165

[B27] Mitschke J Georg J Scholz I Sharma CM Dienst D Bantscheff J Voss B Steglich C Wilde A Vogel J Hess WR An experimentally anchored map of transcriptional start sites in the model cyanobacterium Synechocystis sp. PCC6803 Proc Natl Acad Sci USA 2011 108 2124 2129 10.1073/pnas.1015154108 21245330 PMC3033270

[B28] Koide T Reiss DJ Bare JC Pang WL Facciotti MT Schmid AK Pan M Marzolf B Van PT Lo FY Prevalence of transcription promoters within archaeal operons and coding sequences Mol Syst Biol 2009 5 285 19536208 10.1038/msb.2009.42 PMC2710873

[B29] Hovik H Yu WH Olsen I Chen T Comprehensive transcriptome analysis of the periodontopathogenic bacterium Porphyromonas gingivalis W83 J Bacteriol 2012 194 100 114 10.1128/JB.06385-11 22037400 PMC3256594

[B30] Rasmussen S Nielsen HB Jarmer H The transcriptionally active regions in the genome of Bacillus subtilis Mol Microbiol 2009 73 1043 1057 10.1111/j.1365-2958.2009.06830.x 19682248 PMC2784878

[B31] Perkins TT Kingsley RA Fookes MC Gardner PP James KD Yu L Assefa SA He M Croucher NJ Pickard DJ A strand-specific RNA-Seq analysis of the transcriptome of the typhoid bacillus Salmonella typhi PLoS Genet 2009 5 e1000569 10.1371/journal.pgen.1000569 19609351 PMC2704369

[B32] Yoder-Himes DR Chain PS Zhu Y Wurtzel O Rubin EM Tiedje JM Sorek R Mapping the Burkholderia cenocepacia niche response via high-throughput sequencing Proc Natl Acad Sci USA 2009 106 3976 3981 10.1073/pnas.0813403106 19234113 PMC2645912

[B33] McGrath PT Lee H Zhang L Iniesta AA Hottes AK Tan MH Hillson NJ Hu P Shapiro L McAdams HH High-throughput identification of transcription start sites, conserved promoter motifs and predicted regulons Nat Biotechnol 2007 25 584 592 10.1038/nbt1294 17401361

[B34] Lasa I Toledo-Arana A Dobin A Villanueva M de los Mozos IR Vergara-Irigaray M Segura V Fagegaltier D Penades JR Valle J Genome-wide antisense transcription drives mRNA processing in bacteria Proc Natl Acad Sci USA 2011 108 20172 20177 10.1073/pnas.1113521108 22123973 PMC3250193

[B35] Mandlik A Livny J Robins WP Ritchie JM Mekalanos JJ Waldor MK RNA-Seq-based monitoring of infection-linked changes in Vibrio cholerae gene expression Cell Host Microbe 2011 10 165 174 10.1016/j.chom.2011.07.007 21843873 PMC3166260

[B36] Albrecht M Sharma CM Reinhardt R Vogel J Rudel T Deep sequencing-based discovery of the Chlamydia trachomatis transcriptome Nucleic Acids Res 2010 38 868 877 10.1093/nar/gkp1032 19923228 PMC2817459

[B37] Albrecht M Sharma CM Dittrich MT Muller T Reinhardt R Vogel J Rudel T The transcriptional landscape of Chlamydia pneumoniae Genome Biol 2011 12 R98 10.1186/gb-2011-12-10-r98 21989159 PMC3333780

[B38] Wang Y Li X Mao Y Blaschek HP Single-nucleotide resolution analysis of the transcriptome structure of Clostridium beijerinckii NCIMB 8052 using RNA-Seq BMC Genomics 2011 12 479 10.1186/1471-2164-12-479 21962126 PMC3271303

[B39] Toledo-Arana A Dussurget O Nikitas G Sesto N Guet-Revillet H Balestrino D Loh E Gripenland J Tiensuu T Vaitkevicius K The Listeria transcriptional landscape from saprophytism to virulence Nature 2009 459 950 956 10.1038/nature08080 19448609

[B40] Flaherty BL Van Nieuwerburgh F Head SR Golden JW Directional RNA deep sequencing sheds new light on the transcriptional response of Anabaena sp. strain PCC 7120 to combined-nitrogen deprivation BMC Genomics 2011 12 332 10.1186/1471-2164-12-332 21711558 PMC3141674

[B41] Vijayan V Jain IH O’Shea EK A high resolution map of a cyanobacterial transcriptome Genome Biol 2011 12 R47 10.1186/gb-2011-12-5-r47 21612627 PMC3219970

[B42] Wurtzel O Sapra R Chen F Zhu Y Simmons BA Sorek R A single-base resolution map of an archaeal transcriptome Genome Res 2010 20 133 141 10.1101/gr.100396.109 19884261 PMC2798825

[B43] Selinger DW Cheung KJ Mei R Johansson EM Richmond CS Blattner FR Lockhart DJ Church GM RNA expression analysis using a 30 base pair resolution Escherichia coli genome array Nat Biotechnol 2000 18 1262 1268 10.1038/82367 11101804

[B44] Dornenburg JE Devita AM Palumbo MJ Wade JT Widespread antisense transcription in Escherichia coli MBio 2010 1 pii: e00024-10 10.1128/mBio.00024-10 PMC2912661 20689751

[B45] Neidhardt FC Curtiss R III INgraham JL Lin ECC Low KB Magasanik B Reznikoff WS Riley M Schaechter M Umbarger HE EcoSal : Escherichia coli and Salmonella : cellular and molecular biology 2002 Washington D.C.: ASM Press

[B46] Karp PD Riley M Saier M Paulsen IT Collado-Vides J Paley SM Pellegrini-Toole A Bonavides C Gama-Castro S The EcoCyc database Nucleic Acids Res 2002 30 56 58 10.1093/nar/30.1.56 11752253 PMC99147

[B47] Resendis-Antonio O Freyre-Gonzalez JA Menchaca-Mendez R Gutierrez-Rios RM Martinez-Antonio A Avila-Sanchez C Collado-Vides J Modular analysis of the transcriptional regulatory network of E. coli Trends Genet 2005 21 16 20 10.1016/j.tig.2004.11.010 15680508

[B48] Busby S Ebright RH Promoter structure, promoter recognition, and transcription activation in prokaryotes Cell 1994 79 743 746 8001112 10.1016/0092-8674(94)90063-9

[B49] Browning DF Busbym SJW The regulation of bacterial transcription initiation Nat Rev Microbiol 2004 2 57 65 10.1038/nrmicro787 15035009

[B50] Riley M Abe T Arnaud MB Berlyn MK Blattner FR Chaudhuri RR Glasner JD Horiuchi T Keseler IM Kosuge T Escherichia coli K-12: a cooperatively developed annotation snapshot–2005 Nucleic Acids Res 2006 34 1 9 10.1093/nar/gkj405 16397293 PMC1325200

[B51] Karp PD Keseler IM Shearer A Latendresse M Krummenacker M Paley SM Paulsen I Collado-Vides J Gama-Castro S Peralta-Gil M Multidimensional annotation of the Escherichia coli K-12 genome Nucleic Acids Res 2007 35 7577 7590 10.1093/nar/gkm740 17940092 PMC2190727

[B52] Blattner FR Plunkett G 3rd Bloch CA Perna NT Burland V Riley M Collado-Vides J Glasner JD Rode CK Mayhew GF The complete genome sequence of Escherichia coli K-12 Science 1997 277 1453 1462 10.1126/science.277.5331.1453 9278503

[B53] Gama-Castro S Salgado H Peralta-Gil M Santos-Zavaleta A Muniz-Rascado L Solano-Lira H Jimenez-Jacinto V Weiss V Garcia-Sotelo JS Lopez-Fuentes A RegulonDB version 7.0: transcriptional regulation of Escherichia coli K-12 integrated within genetic sensory response units (Gensor Units) Nucleic Acids Res 2011 39 D98 105 10.1093/nar/gkq1110 21051347 PMC3013702

[B54] Hershberg R Altuvia S Margalit H A survey of small RNA-encoding genes in Escherichia coli Nucleic Acids Res 2003 31 1813 1820 10.1093/nar/gkg297 12654996 PMC152812

[B55] Gottesman S Storz G Bacterial small RNA regulators: versatile roles and rapidly evolving variations Cold Spring Harb Perspect Biol 2011 3 pii: a003798 10.1101/cshperspect.a003798 PMC3225950 20980440

[B56] Storz G Vogel J Wassarman KM Regulation by small RNAs in bacteria: expanding frontiers Mol Cell 2011 43 880 891 10.1016/j.molcel.2011.08.022 21925377 PMC3176440

[B57] Cho BK Zengler K Qiu Y Park YS Knight EM Barrett CL Gao Y Palsson BO The transcription unit architecture of the Escherichia coli genome Nat Biotechnol 2009 27 1043 1049 10.1038/nbt.1582 19881496 PMC3832199

[B58] Mendoza-Vargas A Olvera L Olvera M Grande R Vega-Alvarado L Taboada B Jimenez-Jacinto V Salgado H Juarez K Contreras-Moreira B Genome-wide identification of transcription start sites, promoters and transcription factor binding sites in E. coli PLoS One 2009 4 e7526 10.1371/journal.pone.0007526 19838305 PMC2760140

[B59] Wang Z Gerstein M Snyder M RNA-Seq: a revolutionary tool for transcriptomics Nat Rev Genet 2009 10 57 63 10.1038/nrg2484 19015660 PMC2949280

[B60] Vivancos AP Guell M Dohm JC Serrano L Himmelbauer H Strand-specific deep sequencing of the transcriptome Genome Res 2010 20 989 999 10.1101/gr.094318.109 20519413 PMC2892100

[B61] Levin JZ Yassour M Adiconis X Nusbaum C Thompson DA Friedman N Gnirke A Regev A Comprehensive comparative analysis of strand-specific RNA sequencing methods Nat Methods 2010 7 709 715 10.1038/nmeth.1491 20711195 PMC3005310

[B62] Mortazavi A Williams BA McCue K Schaeffer L Wold B Mapping and quantifying mammalian transcriptomes by RNA-Seq Nat Methods 2008 5 621 628 10.1038/nmeth.1226 18516045 PMC13303166

[B63] Roberts A Trapnell C Donaghey J Rinn JL Pachter L Improving RNA-Seq expression estimates by correcting for fragment bias Genome Biol 2011 12 R22 10.1186/gb-2011-12-3-r22 21410973 PMC3129672

[B64] Cheung MS Down TA Latorre I Ahringer J Systematic bias in high-throughput sequencing data and its correction by BEADS Nucleic Acids Res 2011 39 e103 10.1093/nar/gkr425 21646344 PMC3159482

[B65] Sendler E Johnson GD Krawetz SA Local and global factors affecting RNA sequencing analysis Anal Biochem 2011 419 317 322 10.1016/j.ab.2011.08.013 21889483

[B66] Wu Z Wang X Zhang X Using non-uniform read distribution models to improve isoform expression inference in RNA-Seq Bioinformatics 2011 27 502 508 10.1093/bioinformatics/btq696 21169371

[B67] Li J Jiang H Wong WH Modeling non-uniformity in short-read rates in RNA-Seq data Genome Biol 2010 11 R50 10.1186/gb-2010-11-5-r50 20459815 PMC2898062

[B68] Pop M Genome assembly reborn: recent computational challenges Brief Bioinform 2009 10 354 366 10.1093/bib/bbp026 19482960 PMC2691937

[B69] Flicek P Birney E Sense from sequence reads: methods for alignment and assembly Nat Methods 2009 6 S6 S12 10.1038/nmeth.1376 19844229

[B70] Martin JA Wang Z Next-generation transcriptome assembly Nat Rev Genet 2011 12 671 682 10.1038/nrg3068 21897427

[B71] Trapnell C Williams BA Pertea G Mortazavi A Kwan G van Baren MJ Salzberg SL Wold BJ Pachter L Transcript assembly and quantification by RNA-Seq reveals unannotated transcripts and isoform switching during cell differentiation Nat Biotechnol 2010 28 511 515 10.1038/nbt.1621 20436464 PMC3146043

[B72] Ciesiolka J Michalowski D Wrzesinski J Krajewski J Krzyzosiak WJ Patterns of cleavages induced by lead ions in defined RNA secondary structure motifs J Mol Biol 1998 275 211 220 10.1006/jmbi.1997.1462 9466904

[B73] Hansen KD Brenner SE Dudoit S Biases in Illumina transcriptome sequencing caused by random hexamer priming Nucleic Acids Res 2010 38 e131 10.1093/nar/gkq224 20395217 PMC2896536

[B74] Hafner M Renwick N Brown M Mihailovic A Holoch D Lin C Pena JT Nusbaum JD Morozov P Ludwig J RNA-ligase-dependent biases in miRNA representation in deep-sequenced small RNA cDNA libraries RNA 2011 17 1697 1712 10.1261/rna.2799511 21775473 PMC3162335

[B75] Zhuang F Fuchs RT Sun Z Zheng Y Robb GB Structural bias in T4 RNA ligase-mediated 3’-adapter ligation Nucleic Acids Res 2012 40 e54 10.1093/nar/gkr1263 22241775 PMC3326334

[B76] Jayaprakash AD Jabado O Brown BD Sachidanandam R Identification and remediation of biases in the activity of RNA ligases in small-RNA deep sequencing Nucleic Acids Res 2011 39 e141 10.1093/nar/gkr693 21890899 PMC3241666

[B77] Risso D Schwartz K Sherlock G Dudoit S GC-content normalization for RNA-Seq data BMC Bioinformatics 2011 12 480 10.1186/1471-2105-12-480 22177264 PMC3315510

[B78] Benjamini Y Speed TP Summarizing and correcting the GC content bias in high-throughput sequencing Nucleic Acids Res 2012 40 e72 10.1093/nar/gks001 22323520 PMC3378858

[B79] Aird D Ross MG Chen WS Danielsson M Fennell T Russ C Jaffe DB Nusbaum C Gnirke A Analyzing and minimizing PCR amplification bias in Illumina sequencing libraries Genome Biol 2011 12 R18 10.1186/gb-2011-12-2-r18 21338519 PMC3188800

[B80] Minoche AE Dohm JC Himmelbauer H Evaluation of genomic high-throughput sequencing data generated on Illumina HiSeq and genome analyzer systems Genome Biol 2011 12 R112 10.1186/gb-2011-12-11-r112 22067484 PMC3334598

[B81] Nakamura K Oshima T Morimoto T Ikeda S Yoshikawa H Shiwa Y Ishikawa S Linak MC Hirai A Takahashi H Sequence-specific error profile of Illumina sequencers Nucleic Acids Res 2011 39 e90 10.1093/nar/gkr344 21576222 PMC3141275

[B82] Mamanova L Andrews RM James KD Sheridan EM Ellis PD Langford CF Ost TW Collins JE Turner DJ FRT-seq: amplification-free, strand-specific transcriptome sequencing Nat Methods 2010 7 130 132 10.1038/nmeth.1417 20081834 PMC2861772

[B83] Lipson D Raz T Kieu A Jones DR Giladi E Thayer E Thompson JF Letovsky S Milos P Causey M Quantification of the yeast transcriptome by single-molecule sequencing Nat Biotechnol 2009 27 652 658 10.1038/nbt.1551 19581875

[B84] Raz T Causey M Jones DR Kieu A Letovsky S Lipson D Thayer E Thompson JF Milos PM RNA sequencing and quantitation using the Helicos Genetic Analysis System Methods Mol Biol 2011 733 37 49 10.1007/978-1-61779-089-8_3 21431761

[B85] Kent WJ BLAT–the BLAST-like alignment tool Genome Res 2002 12 656 664 11932250 10.1101/gr.229202 PMC187518

[B86] Trapnell C Pachter L Salzberg SL TopHat: discovering splice junctions with RNA-Seq Bioinformatics 2009 25 1105 1111 10.1093/bioinformatics/btp120 19289445 PMC2672628

[B87] Langmead B Trapnell C Pop M Salzberg SL Ultrafast and memory-efficient alignment of short DNA sequences to the human genome Genome Biol 2009 10 R25 10.1186/gb-2009-10-3-r25 19261174 PMC2690996

[B88] Guttman M Garber M Levin JZ Donaghey J Robinson J Adiconis X Fan L Koziol MJ Gnirke A Nusbaum C Ab initio reconstruction of cell type-specific transcriptomes in mouse reveals the conserved multi-exonic structure of lincRNAs Nat Biotechnol 2010 28 503 510 10.1038/nbt.1633 20436462 PMC2868100

[B89] Grabherr MG Haas BJ Yassour M Levin JZ Thompson DA Amit I Adiconis X Fan L Raychowdhury R Zeng Q Full-length transcriptome assembly from RNA-Seq data without a reference genome Nat Biotechnol 2011 29 644 652 10.1038/nbt.1883 21572440 PMC3571712

[B90] Schulz MH Zerbino DR Vingron M Birney E Oases: robust de novo RNA-seq assembly across the dynamic range of expression levels Bioinformatics 2012 28 1086 1092 10.1093/bioinformatics/bts094 22368243 PMC3324515

[B91] Robertson G Schein J Chiu R Corbett R Field M Jackman SD Mungall K Lee S Okada HM Qian JQ De novo assembly and analysis of RNA-seq data Nat Methods 2010 7 909 912 10.1038/nmeth.1517 20935650

[B92] Martin J Bruno VM Fang Z Meng X Blow M Zhang T Sherlock G Snyder M Wang Z Rnnotator: an automated de novo transcriptome assembly pipeline from stranded RNA-Seq reads BMC Genomics 2010 11 663 10.1186/1471-2164-11-663 21106091 PMC3152782

[B93] Surget-Groba Y Montoya-Burgos JI Optimization of de novo transcriptome assembly from next-generation sequencing data Genome Res 2010 20 1432 1440 10.1101/gr.103846.109 20693479 PMC2945192

[B94] Martin J Zhu W Passalacqua KD Bergman N Borodovsky M Bacillus anthracis genome organization in light of whole transcriptome sequencing BMC Bioinformatics 2010 11 Suppl 3 S10 10.1186/1471-2105-11-S3-S10 20438648 PMC2863060

[B95] Nagalakshmi U Wang Z Waern K Shou C Raha D Gerstein M Snyder M The transcriptional landscape of the yeast genome defined by RNA sequencing Science 2008 320 1344 1349 10.1126/science.1158441 18451266 PMC2951732

[B96] Bailey TL Elkan C Fitting a mixture model by expectation maximization to discover motifs in biopolymers Proc Int Conf Intell Syst Mol Biol 1994 2 28 36 7584402

[B97] Yus E Guell M Vivancos AP Chen WH Lluch-Senar M Delgado J Gavin AC Bork P Serrano L Transcription start site associated RNAs in bacteria Mol Syst Biol 2012 8 585 22617959 10.1038/msb.2012.16 PMC3377991

[B98] Makino K Kim SK Shinagawa H Amemura M Nakata A Molecular analysis of the cryptic and functional phn operons for phosphonate use in Escherichia coli K-12 J Bacteriol 1991 173 2665 2672 1840580 10.1128/jb.173.8.2665-2672.1991 PMC207835

[B99] Hove-Jensen B Rosenkrantz TJ Zechel DL Willemoes M Accumulation of intermediates of the carbon-phosphorus lyase pathway for phosphonate degradation in phn mutants of Escherichia coli J Bacteriol 2010 192 370 374 10.1128/JB.01131-09 19854894 PMC2798254

[B100] Iqbal S Parker G Davidson H Moslehi-Rahmani E Robson RL Reversible phase variation in the phnE gene, which is required for phosphonate metabolism in Escherichia coli K-12 J Bacteriol 2004 186 6118 6123 10.1128/JB.186.18.6118-6123.2004 15342581 PMC515159

[B101] Jochimsen B Lolle S McSorley FR Nabi M Stougaard J Zechel DL Hove-Jensen B Five phosphonate operon gene products as components of a multi-subunit complex of the carbon-phosphorus lyase pathway Proc Natl Acad Sci USA 2011 108 11393 11398 10.1073/pnas.1104922108 21705661 PMC3136323

[B102] Chen CM Ye QZ Zhu ZM Wanner BL Walsh CT Molecular biology of carbon-phosphorus bond cleavage. Cloning and sequencing of the phn (psiD) genes involved in alkylphosphonate uptake and C-P lyase activity in Escherichia coli B J Biol Chem 1990 265 4461 4471 2155230

[B103] Metcalf WW Wanner BL Evidence for a fourteen-gene, phnC to phnP locus for phosphonate metabolism in Escherichia coli Gene 1993 129 27 32 10.1016/0378-1119(93)90692-V 8335257

[B104] Kononova SV Nesmeyanova MA Phosphonates and their degradation by microorganisms Biochemistry (Mosc) 2002 67 184 195 10.1023/A:1014409929875 11952414

[B105] Shi W Zhou Y Wild J Adler J Gross CA DnaK, DnaJ, and GrpE are required for flagellum synthesis in Escherichia coli J Bacteriol 1992 174 6256 6263 1400176 10.1128/jb.174.19.6256-6263.1992 PMC207695

[B106] Rashid MH Rao NN Kornberg A Inorganic polyphosphate is required for motility of bacterial pathogens J Bacteriol 2000 182 225 227 10.1128/JB.182.1.225-227.2000 10613886 PMC94263

[B107] Nicol JW Helt GA Blanchard SG Jr Raja A Loraine AE The integrated genome browser: free software for distribution and exploration of genome-scale datasets Bioinformatics 2009 25 2730 2731 10.1093/bioinformatics/btp472 19654113 PMC2759552

[B108] Bullard JH Purdom E Hansen KD Dudoit S Evaluation of statistical methods for normalization and differential expression in mRNA-Seq experiments BMC Bioinformatics 2010 11 94 10.1186/1471-2105-11-94 20167110 PMC2838869

[B109] Marioni JC Mason CE Mane SM Stephens M Gilad Y RNA-seq: an assessment of technical reproducibility and comparison with gene expression arrays Genome Res 2008 18 1509 1517 10.1101/gr.079558.108 18550803 PMC2527709

[B110] Jones DC Ruzzo WL Peng X Katze MG A new approach to bias correction in RNA-Seq Bioinformatics 2012 28 921 928 10.1093/bioinformatics/bts055 22285831 PMC3315719

[B111] Srivastava S Chen L A two-parameter generalized Poisson model to improve the analysis of RNA-seq data Nucleic Acids Res 2010 38 e170 10.1093/nar/gkq670 20671027 PMC2943596

[B112] Burge C Karlin S Prediction of complete gene structures in human genomic DNA J Mol Biol 1997 268 78 94 10.1006/jmbi.1997.0951 9149143

[B113] Larsen TS Krogh A EasyGene–a prokaryotic gene finder that ranks ORFs by statistical significance BMC Bioinformatics 2003 4 21 10.1186/1471-2105-4-21 12783628 PMC521197

[B114] Reese MG Kulp D Tammana H Haussler D Genie–gene finding in Drosophila melanogaster Genome Res 2000 10 529 538 10.1101/gr.10.4.529 10779493 PMC310881

[B115] Durbin R Eddy S Krogh A Mitchison G Biological sequence analysis 1998 Cambrage, UK: Cambridge University Press

[B116] Su Z Olman V Mao F Xu Y Comparative genomics analysis of NtcA regulons in cyanobacteria: regulation of nitrogen assimilation and its coupling to photosynthesis Nucleic Acid Res 2005 33 5156 5171 10.1093/nar/gki817 16157864 PMC1214546

[B117] Li S Xu M Su Z Computational analysis of LexA regulons in Cyanobacteria BMC Genomics 2010 11 527 10.1186/1471-2164-11-527 20920248 PMC3091678

