# Supplementary material for: Directional RNA-seq reveals highly complex condition-dependent transcriptomes in E. coli K12 through accurate full-length transcripts assembling
Source: BMC Genomics. 2013 Jul 30;14:520. doi: 10.1186/1471-2164-14-520 (PMC3734233; doi:10.1186/1471-2164-14-520)
Supplement: Additional file 1 — Supporting figures and tables. Figure S1-S10 and Table S1-S10. [file 1471-2164-14-520-S1.docx]

**Supporting Figures and Tables**


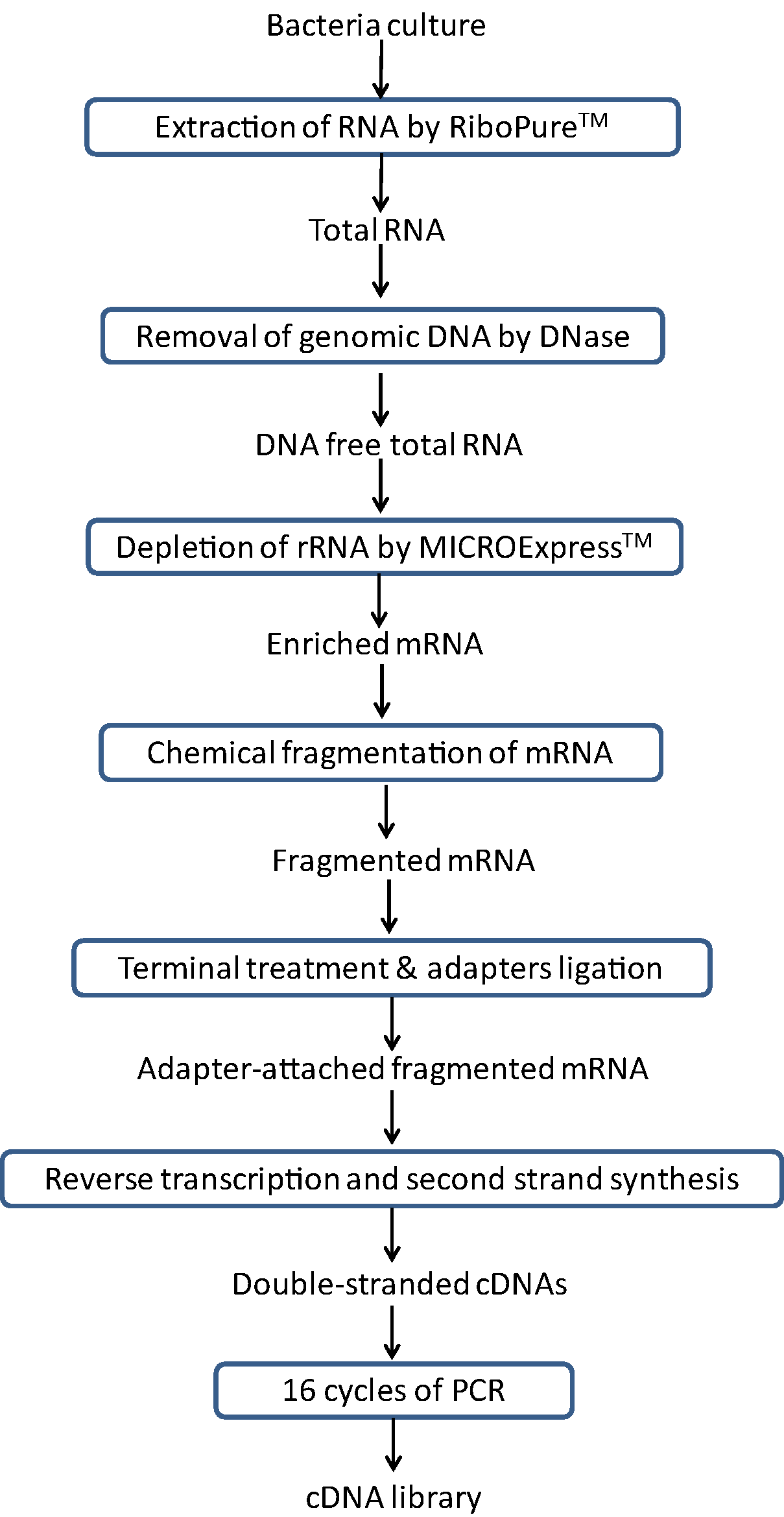


**Figure S1.** Flowchart for the construction of directional RNA-seq libraries.

A

B


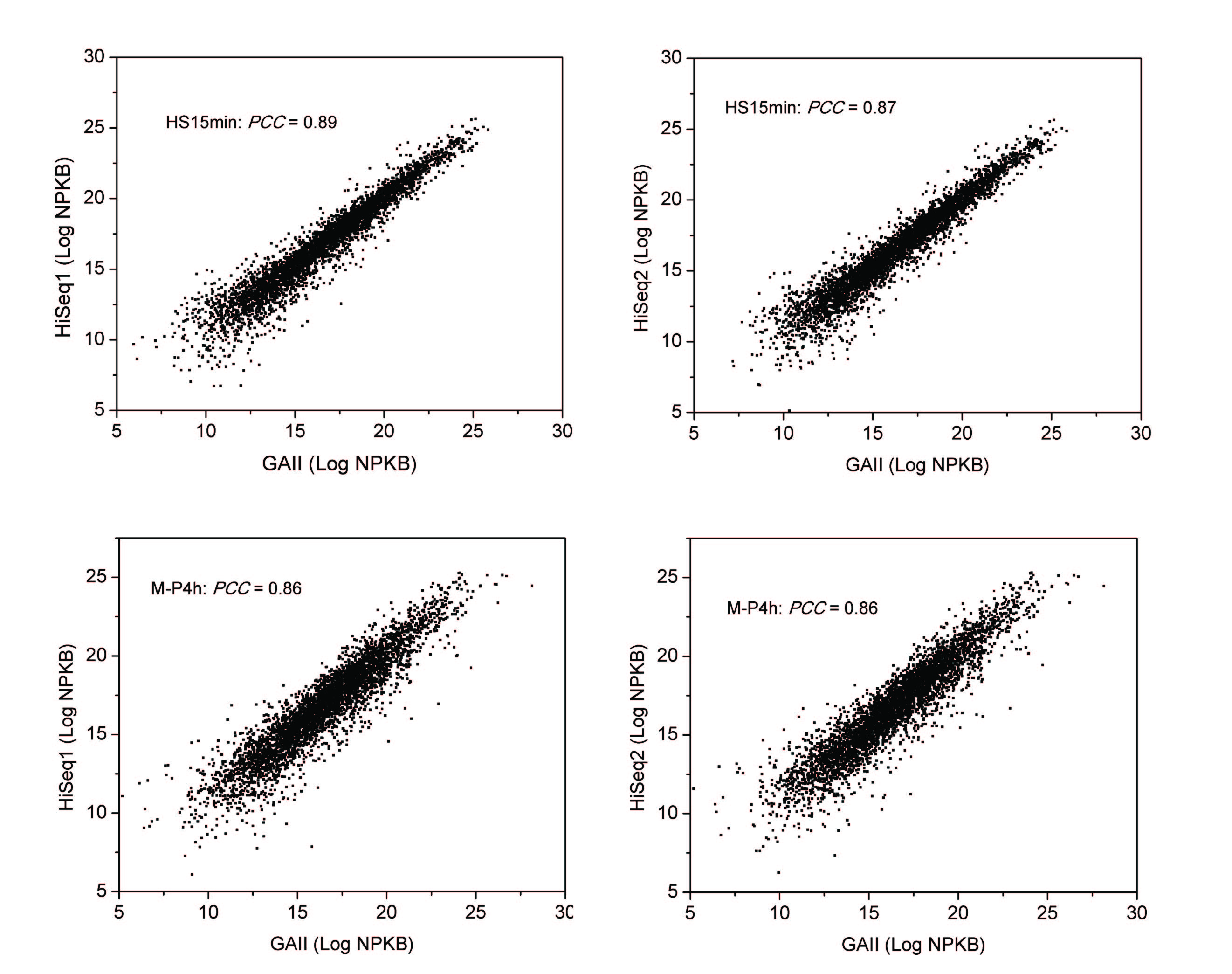


D

C

**Figure S2.** Correlation of expression levels of all the genes between the GAII and HiSeq platforms. Each dot represents a gene. The expression level is log of the NPKB values. A) PCC of expression levels for HS15min between GAII reads and HiSeq reads. B) PCC of expression levels for HS15min between GAII reads and 2^nd^ HiSeq reads. C) PCC of expression levels for M-P4h between GAII reads and HiSeq reads. D) PCC of expression levels for M-P4h between GAII reads and 2^nd^ HiSeq reads. The duplicates for each sample are from the same biological samples sequenced twice using the HiSeq 2000 platform.


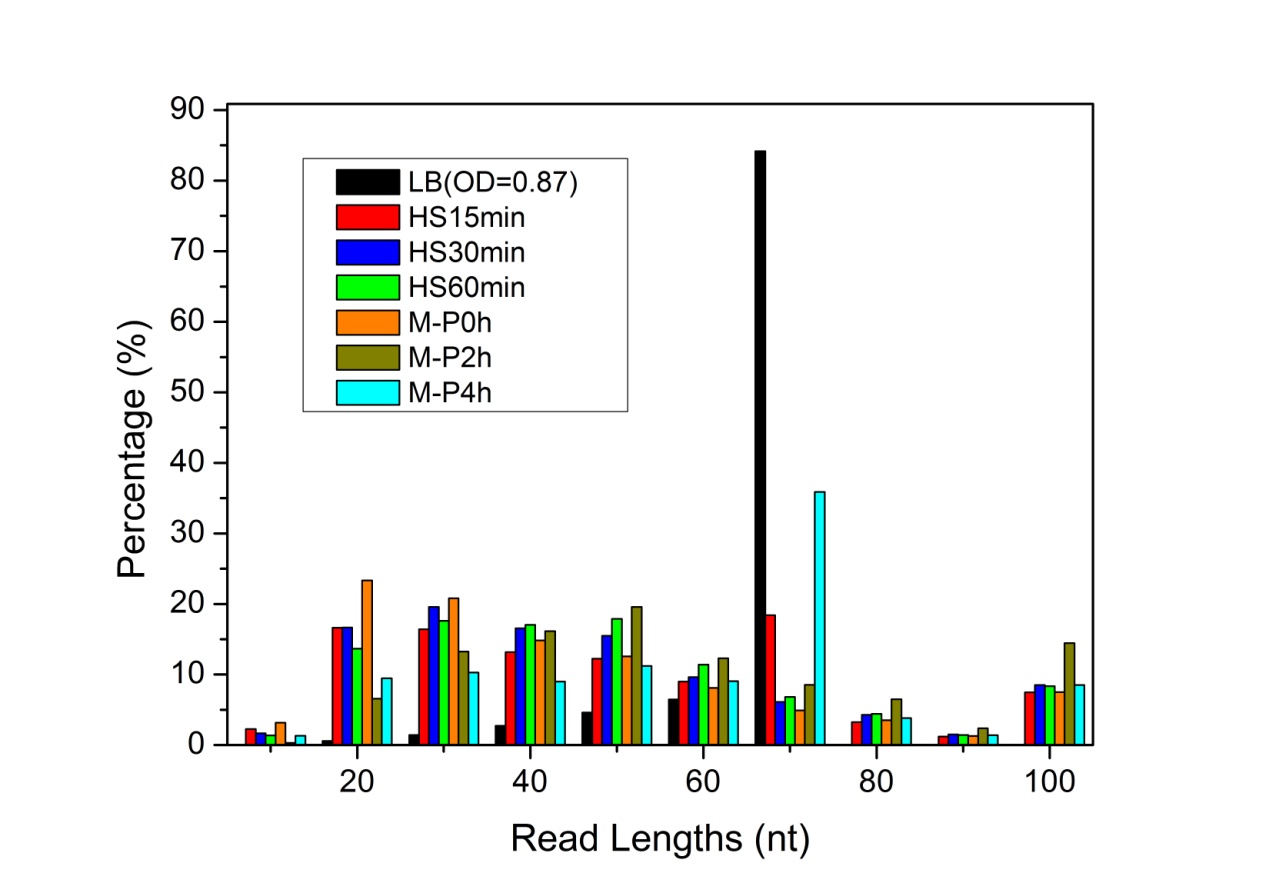


**Figure S3.** Distribution of the genes with more than the indicated percentage of their length covered by at least one read in the samples generated by Vivancos *et. al* [[1](#_ENREF_1)]**.** Less than 60% of genes have over 50% of their length covered by at least one read.

**
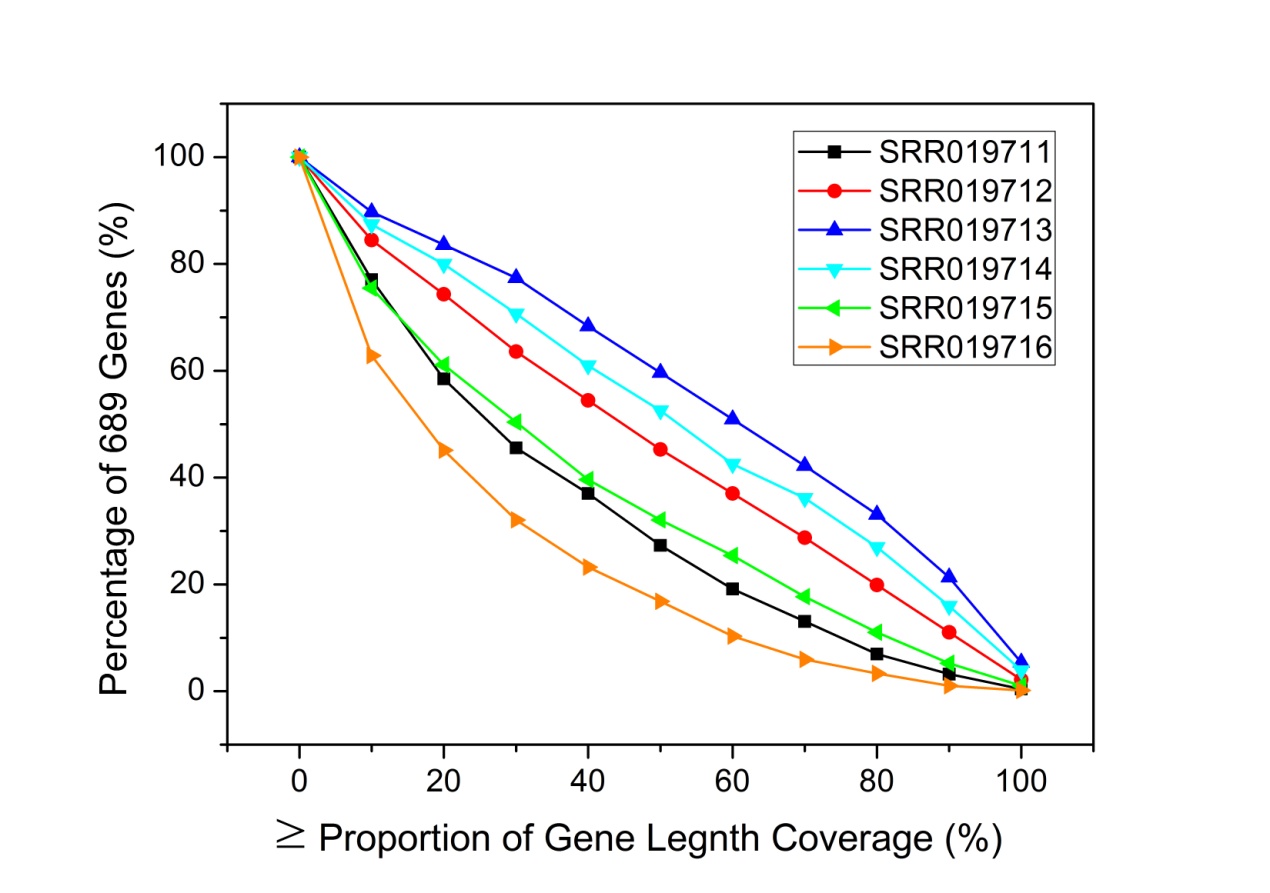
**

**Figure S4.** Distribution of the length of the uniquely mapped reads in the samples.

**
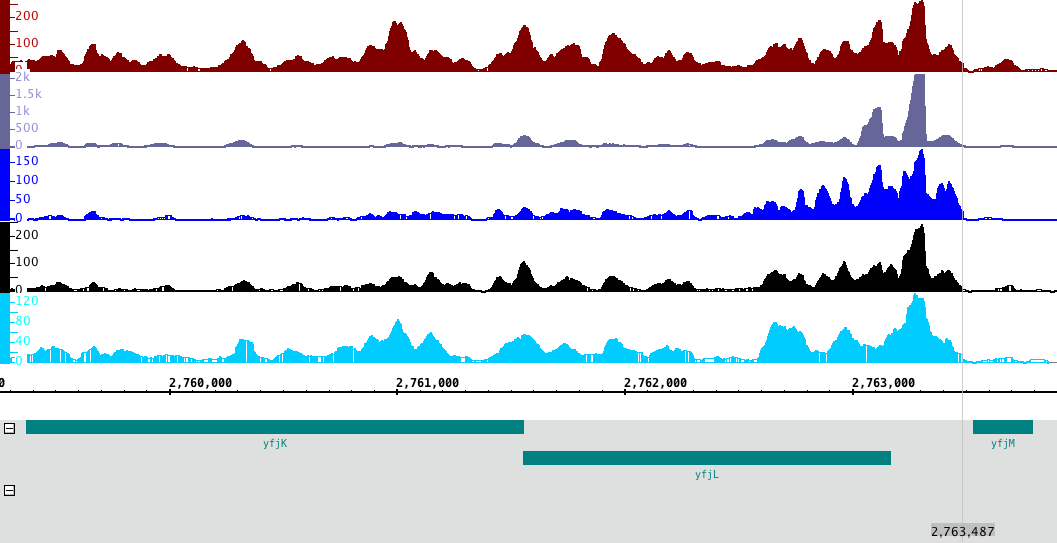
**

M-P4h

M-P2h

M-P0h

HS15min

LB

b2627

b2628

b2629

**Figure S5.** Position-dependent non-uniform read coverage along of the operon b2628-b2627. Not the highly similar patterns of the non-uniform coverage under different culture conditions and growth phases. Although no TSS is documented for this operon in RegulonDB, we identified the position 2,763,486 as the TSS for the operon in *E. coli* K12 in five samples LB, HS15min, M-P0h, M-P2h, and M-P4h.

**
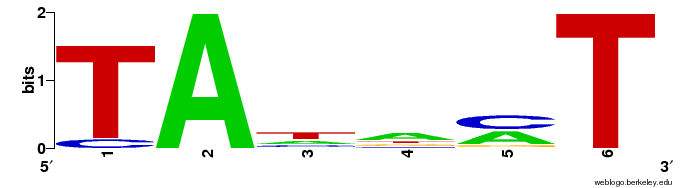
**

A

**
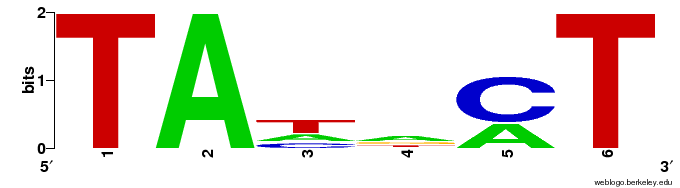
**

B

**
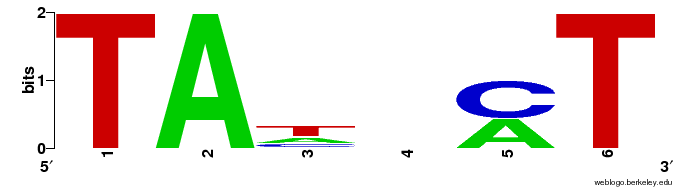
**

C

**Figure S6.** Detected σ^70^ binding sites (Pribnow boxes) in the promoter regions of the known TSSs and predicted TSSs. A) Pribnow box found by MEME [[3](#_ENREF_3)] in 539 of the 1742 known upstream promoter sequences (25nt). B) Pribnow box found in the [-100, 100] regions of the predicted TSSs appearing in multiple samples at p-value ≥ 0.05. C) Pribnow box found on the [-100, 100] regions of the predicted TSSs appearing only in one sample at p-value ≤ 0.05.

**
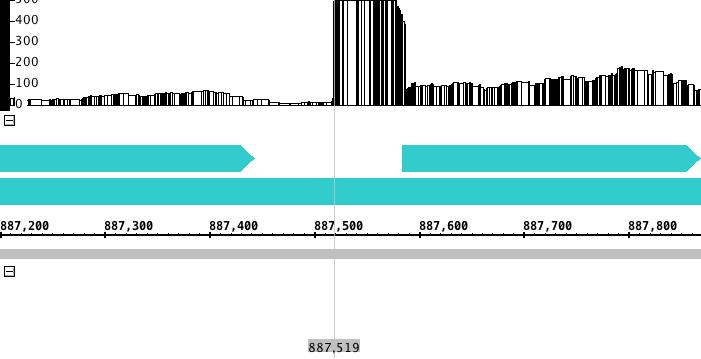
**

SRR031127

HP0834

HP0835

**Figure S7.** Recovery of TSSs in the *H. pylori* data by our algorithm. The light vertical line at position 887,519 indicates the TSS of the gene HP0835 in sample SRR031127 determined by dRNA-seq by Sharma *et. al* [[4](#_ENREF_4)], and our algorithm made the same prediction.

SRR031130

A

**
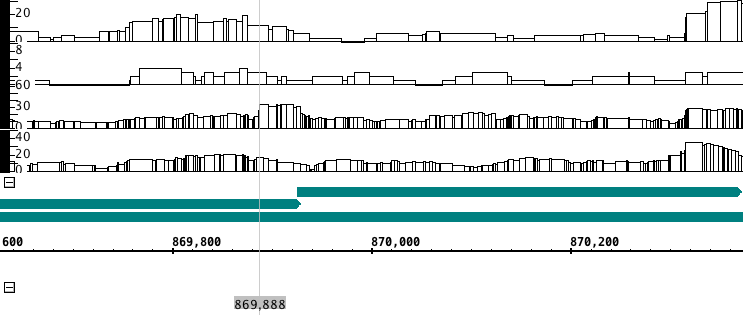
**

SRR031128

SRR031127

SRR031126

HP0186

HP0187

B


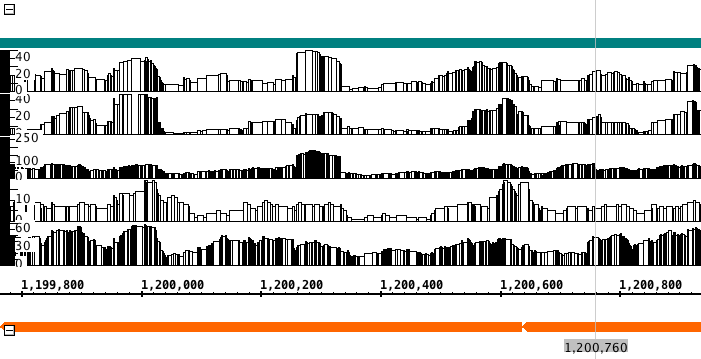


SRR031130

SRR031129

SRR031128

SRR031127

SRR031126

HP1139

HP1138

**Figure S8.** Unrecovered TSSs determined by dRNA-seq by Sharma *et. al* [[4](#_ENREF_4)]. The light vertical lines indicate the positions of the TSSs. A) The determined TSS at 869,888 of the gene HP0187 is located in body of the upstream gene HP0186. B) The determined TSS at 1,200,759 of the gene HP1138 is located in the body of the upstream gene HP1139.

**Figure S9**. **Derivation of transition probabilities**$P_{EE}\text{ and }P_{NN}$. The geometric distribution $\Pr(X=k)=(1-p)^{k}p$ is used to model the number of failures before the first success. The length for the consecutive expression state *E* or non-expression state *N* should follow a geometric distribution. Therefore the probability of staying in the expression state $P_{EE}=1-P_{EN}=\frac{E(X)}{E(X)+1}$, and the probability to transit from the expression state to the non-expression state is $P_{EN}=\frac{1}{E(X)+1}$. Similar results can be derived for$P_{NN}$and$P_{NE}$.

**
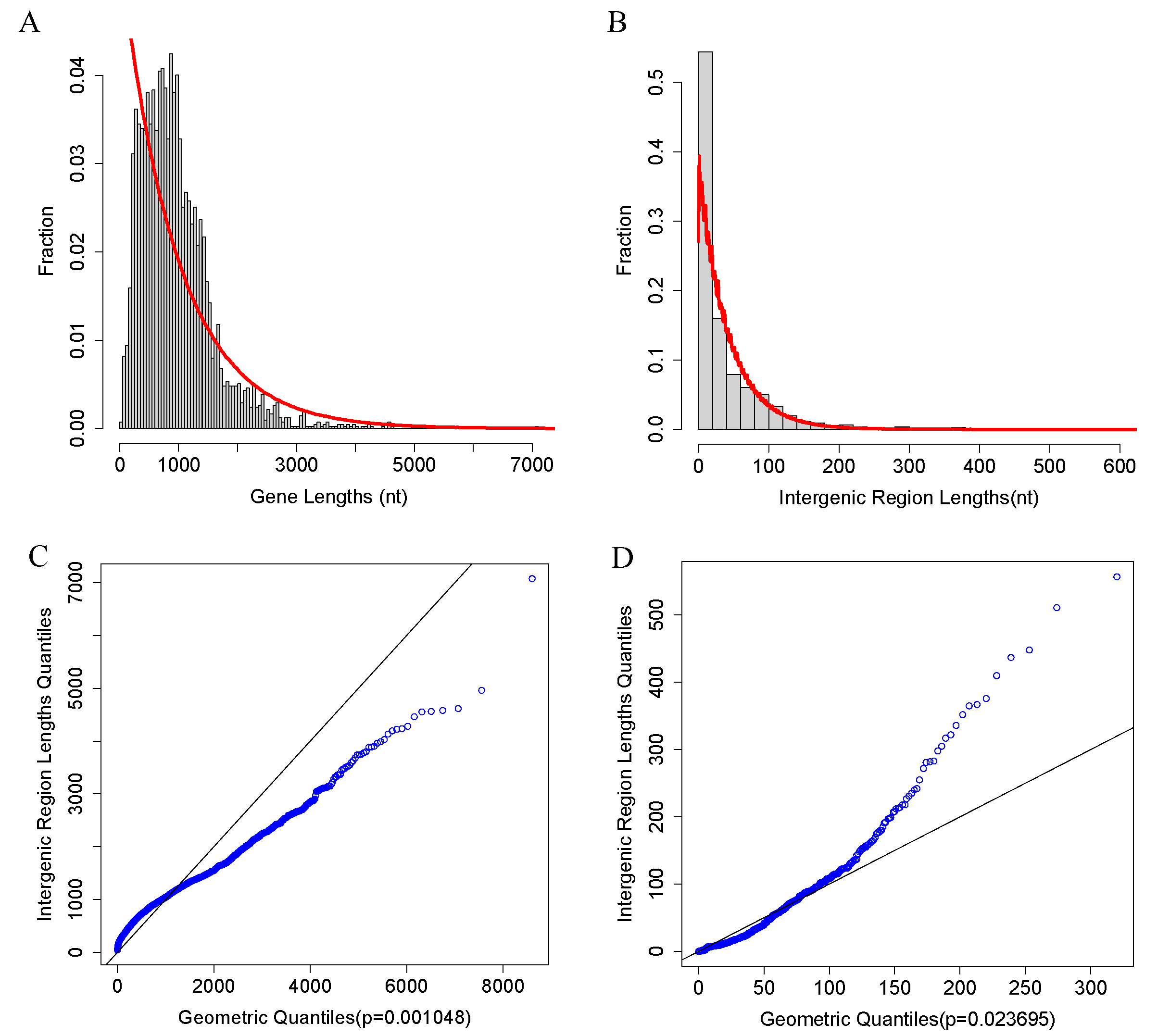
**

**Figure S10**. Distributions of the lengths of ORFs and intergenic regions in the known operons in *E. coli* K12. A: Histogram of the lengths of ORFs (bin size =50nt). The curve is the geometric distribution with the success probability p = 0.0010483 estimated by the maximum likelihood method. B: Histogram of the lengths of intergenic regions within the known operons in ReguonDB (bin size =20nt). The curve is the geometric distribution with the success probability p = 0.0023695 estimated by the maximum likelihood method. C: QQ-plot of the lengths of ORFs against the fitted geometric distribution. D: QQ-plot for the lengths of intergenic regions within the known operons against the fitted geometric distribution. Clearly, unlike the distribution of the lengths of interoperonic regions, the lengths of ORFs cannot be fitted to a geometric distribution.

**Table S1.** Summary of the mapping results

**Table S2.** Effect of sequencing depth on the performance of TruHmm using sample M-P4h as an example

**Table S3.** Performance of TruHmm on the *H. pylori* dataset evaluated based on operon pairs

**Table S4.** Performance of TruHmm on the *H. pylori* dataset of evaluated based on the entire operon structure

**Table S5.** Comparison of the parameters trained on the *E. coli* and *H. pylori* datasets using a window size 11nt and the leave-one-out strategy

**Table S6.**  Specificity of predicted TSSs in the five samples of the *H. pylori* dataset [[4](#_ENREF_4)].

**Table S7**. Summary of assembled operons in the samples

**Table S8**. Reconstruction of alternative *phn* operons

**Table S9**. Reconstruction of alternative *fli* operons

**Table S10.** Proportion of the ORFs and intergenic regions having antisense and non-coding RNA transcriptions

**References**

**1. Vivancos AP, Guell M, Dohm JC, Serrano L, Himmelbauer H: Strand-specific deep sequencing of the transcriptome. *Genome Res* 2010, 20:989-999.**

**2. Gama-Castro S, Jimenez-Jacinto V, Peralta-Gil M, Santos-Zavaleta A, Penaloza-Spinola MI, Contreras-Moreira B, Segura-Salazar J, Muniz-Rascado L, Martinez-Flores I, Salgado H, et al: RegulonDB (version 6.0): gene regulation model of Escherichia coli K-12 beyond transcription, active (experimental) annotated promoters and Textpresso navigation. *Nucleic Acids Res* 2008, 36:D120-124.**

**3. Bailey TL, Elkan C: Fitting a mixture model by expectation maximization to discover motifs in biopolymers. *Proc Int Conf Intell Syst Mol Biol* 1994, 2:28-36.**

**4. Sharma CM, Hoffmann S, Darfeuille F, Reignier J, Findeiss S, Sittka A, Chabas S, Reiche K, Hackermuller J, Reinhardt R, et al: The primary transcriptome of the major human pathogen Helicobacter pylori. *Nature* 2010, 464:250-255.**
